# Developing a Co‐Designed Strategy to Improve Labor Monitoring and Management in India Using the World Health Organization Labour Care Guide: A Mixed‐Methods Formative Study

**DOI:** 10.1111/birt.70004

**Published:** 2025-08-13

**Authors:** Elizabeth Armari, Sunil S. Vernekar, Yeshita Pujar, Veronica Pingray, Fernando Althabe, Luz Gibbons, Mabel Berrueta, Alvaro Ciganda, Rocio Rodriguez, Jayashree Ashok Kumar, Shruti Bhavi Patil, Aravind Karinagannanavar, Raveendra R. Anteen, Pavithra M. R., Savitri Bendigeri, Shukla Shetty, B. Latha, Megha H. M., Suman S. Gaddi, Shaila Chikkagowdra, Bellara Raghavendra, Caroline S. E. Homer, Manjunath Somannavar, Shivaprasad S. Goudar, Joshua P. Vogel

**Affiliations:** ^1^ Women's, Children's and Adolescents' Health Program Burnet Institute Melbourne Victoria Australia; ^2^ Women's and Children's Health Research Unit, Jawaharlal Nehru Medical College KLE Academy of Higher Education and Research Belgaum India; ^3^ Instituto de Efectividad Clínica y Sanitaria (IECS‐CONICET) Buenos Aires Argentina; ^4^ Gadag Institute of Medical Sciences Gcadag India; ^5^ General Hospital, Gokak Belgaum India; ^6^ JJM Medical College Davangere India; ^7^ Ballari Medical College and Research Center (BMCRC) Ballari India

**Keywords:** implementation strategy, labor monitoring, labour care guide

## Abstract

**Introduction:**

Nearly half of all perinatal deaths occur during the intrapartum period due to inadequate labor monitoring and intervention. The partograph, a paper‐based labor monitoring tool, can assist providers in recognizing and acting on early signs of fetal–maternal distress if used effectively. In 2020, the World Health Organization (WHO) developed a “next generation” partograph called the Labour Care Guide. There is limited evidence of how to optimize the use and impact of this new tool. This study describes the development of a co‐designed LCG implementation strategy in Karnataka, India.

**Methods:**

A targeted literature review, primary research across four public maternity hospitals (provider survey and facility assessment), and a 2‐day co‐design workshop with stakeholders were conducted. Findings were mapped to six target behaviors using the Theoretical Domains Framework (TDF) and the Capability, Opportunity, and Motivation‐Behavior (COM‐B) model to identify potential barriers and facilitators to LCG use. Consultations with local stakeholders explored these factors, and a 1‐week pilot informed final refinements of the strategy.

**Results:**

The LCG implementation strategy comprised an evidence‐based provider training program centered on “low dose, high frequency” principles, and monthly audit and feedback cycles, which in turn, relies on an enabling practice environment (supportive national policy frameworks, facility‐level guidelines, external partnerships, senior support, defining provider roles and expectations and adequate equipment and resources) to support its implementation.

**Conclusion:**

Effective use of the LCG needs a robust, context‐sensitive implementation strategy. We present the first evidence‐based, co‐designed LCG implementation strategy which can be used to support LCG dissemination and uptake.

## Graphical abstract

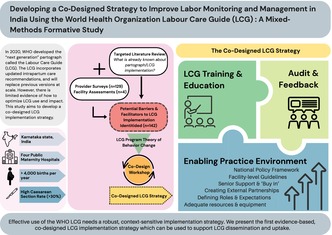

## 1 Background

Maternal and neonatal mortality rates and rates of stillbirth reflect profound global health inequalities [1, 2]. Of the estimated 287,000 maternal deaths, 2.5 million neonatal deaths, and 1.9 million stillbirths recorded each year, the majority occurred in low‐and‐middle‐income countries (LMICs), accounting for 94%, 57%, and 84% of these deaths, respectively [3, 4]. Nearly half of all perinatal deaths occur during the intrapartum period, primarily due to a lack of timely intervention because of ineffective or absent monitoring during labor and birth [5, 6, 7]. High‐quality intrapartum care delivered by skilled health personnel trained to recognize and act on early signs of fetal or maternal distress is the key to preventing these deaths [2, 8].

The partograph (or partogram) is a paper‐based labor monitoring tool which prompts skilled birth attendants to regularly assess and document key parameters of labor progress and maternal‐fetal wellbeing, such as cervical dilation and fetal heart rate. When used prospectively, abnormalities can be detected early, allowing for timely intervention to avoid complications [9]. The World Health Organization (WHO) has recommended the routine use of the partograph since the 1990s, following a large trial of 35,484 women in Southeast Asia which showed significant reductions in prolonged labor, oxytocin augmentation, and stillbirths when the partograph was implemented alongside an intensive labor monitoring training program [10]. However, there have been doubts raised as to whether the partograph is meeting its potential when used in real‐world settings [9, 11, 12, 13].

Partograph use is known to be suboptimal in many settings. High patient loads, inadequate provider training, and ambiguous policy guidance are common barriers [9, 11, 12]. While the partograph appears to be a simple tool, its effectiveness relies on a complex cascade of events which starts with the provider recognizing an abnormality, understanding what to do next, and then having the resources available to intervene appropriately [9, 12]. Providers must use critical decision‐making skills to ensure birth interventions, such as caesarean section (CS), are neither underused nor overused [4, 14]. Historically, WHO partographs have not monitored elements of supportive care which influence women's health outcomes and their experiences of care, such as labor companionship and pain relief.

Over the last three decades, India has made significant improvements in reducing national maternal and infant mortality—a 77% decline in the maternal mortality ratio (MMR) was achieved between 1990 and 2016 [4]. This has largely been driven by rising institutional birth rates nationwide, from 38.7% in 2005 to 88.6% in 2021 [15, 16]. Despite these gains, one‐third of stillbirths in India occur in the intrapartum period, and rates of intrapartum interventions, such as CS, are above recommended thresholds in many hospitals [15, 17].

In 2018, WHO released updated intrapartum care recommendations emphasizing both the provision and experiences of care as critical elements of high‐quality maternity services [14]. To translate these recommendations into practice, WHO published the “next generation” partograph, the Labour Care Guide (LCG), in 2020. The LCG represents a significant departure from previous designs and requires a new approach to intrapartum monitoring and care decisions. It does not include “action” or “alert” lines in favor of individualized labor progress and includes prompts to regularly offer supportive care practices (labor companionship, birth position of choice, mobility in labor and pain relief). Considering the historical challenges to partograph implementation, the LCG's introduction is an opportunity to re‐examine how intrapartum care can be optimized.

This mixed‐methods formative research study aimed to develop a co‐designed LCG implementation strategy (referred to hereon as the “LCG strategy”) to optimize LCG use in four maternity hospitals in Karnataka, India. A key objective was to identify barriers and facilitators to current partograph/LCG use and apply theoretical frameworks to identify which key behaviors need to change at individual, facility, and health system levels to support LCG implementation. Local stakeholders were engaged throughout the design process to ensure sensitivity to context and system fit. The LCG strategy developed in the present study was subsequently evaluated in a stepped‐wedge, cluster‐randomized pilot trial, which found a 5.5% absolute reduction in the CS rate among women in Robson Group 1 (45.2% versus 39.7%) with no maternal or newborn harms [18]. In light of these promising effects, this paper explores how the LCG strategy was developed using an evidence‐based, theory‐informed approach.

## 2 Methods

### 2.1 Study Setting

This study was conducted in four public maternity hospitals (three tertiary, one secondary) in Karnataka State between January 30, 2021 and June 30, 2021. Each hospital manages at least 4000 birthing women each year. The hospitals were purposively selected for their high CS rates (> 30%) and capacity to provide comprehensive emergency obstetric care. All hospitals had either completed or were working towards accreditation under the Government of India's Labour Room Quality Initiative (LaQshya) [19]. The recommendations within the LaQysha initiative aligned closely with the 2018 WHO intrapartum care recommendations [14], including routine use of a partograph. During this study, the WHO simplified partograph was in routine use in all four hospitals.

### 2.2 Overview of Study Design

We conducted a mixed‐methods formative study to develop a theory‐based, evidence‐informed strategy to support the transition to LCG use and optimize its implementation. As the LCG is a novel tool, there is little direct evidence on its use and the conditions required to support it. Hence, we considered factors broadly affecting partograph implementation. To do this, our study objectives included: (1) a targeted literature review to identify what is already known about partograph/LCG use and its implementation; (2) primary research (provider surveys and facility assessments) at each hospital to understand how the WHO partograph is used, current labor monitoring and management practices, and site‐specific barriers or facilitators; (3) a 2‐day intensive co‐design workshop with local stakeholders to review the findings of our primary research and literature review and develop the first iteration of the LCG strategy; and (4) to pilot and refine this LCG strategy.

### 2.3 Study Participants

In public maternity hospitals in India, intrapartum care is provided predominantly by doctors who have completed an additional 3 years of formal training in obstetrics and gynecology (consultants) or are completing their training program (postgraduate doctors). The training curriculum is dictated by the Federation of Obstetrics and Gynecological Societies, India (FOGSI) and is designed, regulated, and standardized by the National Medical Commission (NMC) of India. Postgraduate doctors must complete compulsory labor room placements in 3‐month rotational blocks. Hospitals receive different numbers of postgraduates per rotation, depending on training and workforce needs. In three of the study hospitals, nurses had an auxiliary role and were responsible for completing administrative tasks such as clerking patients. In one hospital (Hospital 4), nurses had been upskilled to provide intrapartum care (including facilitating vaginal births). Midwives were not a recognized cadre in the study hospitals.

### 2.4 Study Procedures

1. *Targeted literature review*: aimed to identify key barriers or facilitators to LCG or partograph implementation as a starting point for our program theory. Sentinel papers or guidelines were identified through PubMed and gray literature searches by two researchers (EA and JV).
2. *Facility assessments*: aimed to examine how intrapartum care was delivered at each hospital, and the current availability of resources, supplies, and equipment. Facility assessment data were collected using a standardized form completed by a study investigator through direct observations and interviews with labor room staff during a single visit (File S1).
3. *Provider survey*: An anonymous, paper‐based survey to explore provider experiences and perceptions of labor monitoring and partograph use. All providers who were currently using the WHO simplified partograph to monitor women in labor, regardless of cadre or seniority, were invited to complete the survey. The survey used a combination of dichotomous, Likert scales, short answer, and multi‐option formats (File S2). Survey responses were entered into REDCap and analyzed using STATA version 14.0.
4. *Co‐design workshop*: This 2‐day workshop was held in person, with some stakeholders joining online due to COVID‐19 travel restrictions. Stakeholders included maternity care providers from the four study hospitals (with a mix of seniority and cadre), state government representatives, and research team members. Group discussions centered on refining our program theory, acceptability, and feasibility of the LCG in study hospitals, and what components of the LCG strategy were essential. Discussions also focused on how to address current deficits in the labor ward environment that might limit LCG use, particularly around supportive care interventions. Field notes and audio recordings of the workshop were collected and analyzed.
5. *Piloting the LCG strategy*: The first LCG strategy iteration was implemented within a nearby tertiary, non‐study hospital for a 1‐week period. Further refinements of the strategy were made based on the pilot and discussions with study stakeholders.

### 2.5 Data Analysis

This study was guided by the Medical Research Council (MRC‐UK) framework for complex interventions [20]. Central to our methodology was the development and refinement of an LCG program theory of behavior change (referred to as “program theory”) by the core research team (EA, JV, VP, SV, YP, SG). We mapped how we expected the LCG to be used, the conditions required for it to be used effectively, and the interactions of these mechanisms within the maternity care environment. This process highlighted six target behaviors for staff to ensure effective LCG implementation: (1) routinely offering supportive care, (2) consistent and prospective LCG use, (3) appropriate clinical decision‐making and use of intrapartum interventions, (4) delivery of woman‐centered, respectful care, (5) labor monitoring education and training, and (6) audit and feedback processes.

Results from the literature review and primary research were tabulated and categorized by two researchers (EA and JV) across these six target behaviors. To explore a broader range of factors influencing these behaviors, we adopted the Theoretical Domains Framework (TDF), which comprises 14 domains of cognitive, affective, social, and environmental influences on behavior [21, 22]. These 14 domains can be further condensed into three overarching constructs essential for behavior change: Capability, Opportunity, and Motivation (the COM‐B model) [21]. By mapping our findings to these two linked frameworks, the key barriers and enablers to LCG implementation emerged, and our program theory was updated accordingly.

The program theory and complete list of identified barriers and facilitators were presented at the co‐design workshop and used as a basis for designing the LCG strategy. Workshop discussions were centered on what factors needed to be prioritized, how to address barriers and enhance facilitators at each site, and the necessary steps required to operationalize these solutions. The program theory was further revised to reflect these discussions, and the components of the LCG strategy emerged from this. The first iteration of the LCG strategy was then piloted in an independent hospital and further refined into the final version.

## 3 Results

**FIGURE 1:**
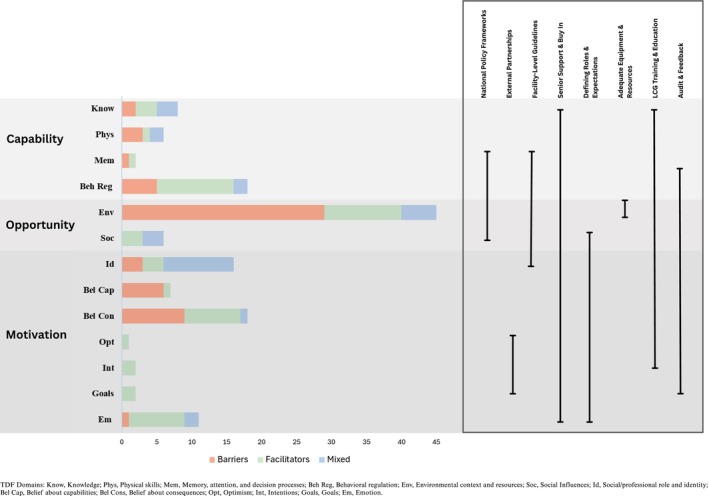
Developing the components of the Labour Care Guide (LCG) implementation strategy using Capability, Opportunity, and Motivation‐Behavior (COM‐B) and Theoretical Domains Framework (TDF) domains. TDF Domains: Know, Knowledge; Phys, Physical skills; Mem, Memory, attention, and decision processes; Beh Reg, Behavioral regulation; Env, Environmental context and resources; Soc, Social Influences; Id, Social/professional role and identity; Bel Cap, Belief about capabilities; Bel Cons, Belief about consequences; Opt, Optimism; Int, Intentions; Goals, Goals; Em, Emotion. [Colour figure can be viewed at wileyonlinelibrary.com]

Key characteristics of study hospitals and survey respondents are presented in Table 1. All eligible providers across the four hospitals completed the survey (*n* = 129), with most respondents being postgraduate doctors (59.7%). We identified 142 barriers and facilitators to current labor monitoring and management (File S3), which were mapped to COM‐B and TDF domains (Figure 1). Of these, almost one‐third related to “environmental context and resources” (*n* = 45). The final version of the LCG strategy was informed by these findings and incorporates targeted optimization of potential barriers whilst leveraging pre‐existing facilitators. A narrative summary of findings for each target behavior is presented below.

**TABLE 1:** Summary of study hospital characteristics and survey respondents.

	Hospital 1	Hospital 2	Hospital 3	Hospital 4
Facility level	Tertiary	Tertiary	Tertiary	Secondary
Number of births^a^	8594	5037	4758	5196
Number of caesarean sections (% of total births)	3667 (42.7%)	2833 (56.2%)	1578 (33.2%)	2367 (45.6%)
LaQysha accreditation obtained	No	No	No	Yes
Number of beds for women in first stage of labor	10	8	5	5
Number of beds for women in second stage of labor	10	8	4	4
Number of surgical theaters for obstetric use	2	2	2	1
WHO simplified partograph completed by:	C	C, PG	C, PG	C, N
Facility‐level guideline mandating routine use of partograph	Yes	No	Yes	Yes
NICU on site	Yes	Yes	Yes	Yes
ICU on site	Yes	No	Yes	No
Blood bank on site	Yes	No	No	Yes
Labor companions permitted during first stage of labor	No	No	Yes	Yes
Labor companions permitted during second stage of labor	No	No	No	Yes
Availability of parenteral opioids fentanyl, pethidine, diamorphine	Yes	Yes	No	No
Availability of tramadol (PO, IV)	Yes	Yes	Yes	Yes
Availability of simple analgesia (paracetamol)	Yes	Yes	Yes	Yes
Birth position of choice offered to women	No	No	Yes	No
Number of total formal training sessions per week	4	3	4	0
Provider attendance at formal training sessions is mandatory	Yes	No	No	Yes
Frequency of pre‐existing audit meetings	Monthly	Every 3 months	Monthly	Monthly
Providers responding to survey (*n*, % of total respondents)				
Number of consultant OBGYN (*n* = 39, 30.2%)	18 (14%)	9 (7%)	8 (6.2%)	4 (3.1%)
Number of postgraduate doctors (*n* = 77, 59.7%)	28 (21.7%)	8 (6.2%)	41 (31.8%)	0 (0%)
Number of nurse/nurse‐midwife (*n* = 13, 10.1%)	0 (0%)	0 (0%)	0 (0%)	13 (10.1%)
Mean years in current role (SD)	5.66 (7.76)	3.35 (6.52)	3.29 (5.75)	11.4 (8.37)

Abbreviations: C, OBGYN consultants; *N*, nurses; PG, postgraduate doctors.

^a^ Number of births for the year 2020.

### 3.1 Routinely Offering Supportive Care

While routine offer of supportive care is mandated within the national policy [19], only one hospital had formally adopted this into local guidelines. Labor companions were only permitted at two hospitals and limited to female‐only relatives or friends. At a third hospital, companions were allowed to stay during active labor but had to leave during the second stage. Some providers expressed concerns about the consequences of introducing labor companionship, believing it could risk their personal safety and compromise patient privacy, and that companions would interfere with patient care. Medications for pain relief were not consistently available—clinicians often saved them for women undergoing an instrumental or CS birth. Although non‐pharmacological pain relief could be facilitated at each hospital (e.g., heat packs, breathing techniques), these options were not prioritized or regularly discussed with women. Overall, mobilization in the early stages of labor was encouraged, but limited privacy and labor room space made this difficult to operationalize. Women only birthed in the supine position in three hospitals, as postgraduate doctors and consultants did not feel adequately trained or confident to offer alternatives. Oral fluid intake was readily encouraged for all laboring women, but food intake was restricted during later stages of labor or for women deemed to be high‐risk of requiring a CS. In general, providers felt they had the skills and knowledge to provide supportive care, but had limited space, medical supplies and workforce to offer it.

### 3.2 Consistent, Prospective Partograph Use

All hospitals had guidelines mandating routine partograph use, but in practice, this was inconsistent and varied. Partographs were often incomplete or filled out retrospectively due to inadequate staffing, high patient loads, and lack of working equipment at the time of clinical assessments (e.g., thermometers or fetal dopplers). Junior doctors (interns and residents) and nurses (except at Hospital 4) were not permitted to fill out the partograph, though they were expected to perform some clinical assessments (e.g., taking maternal blood pressure). Senior staff were usually expected to provide supportive supervision and feedback in real time to ensure the partograph was being used correctly. In practice, senior support and clinical supervision were inconsistent.

### 3.3 Appropriate Clinical Decision Making and Use of Intrapartum Interventions

Most intrapartum decisions were made by the most senior clinician on duty (usually consultants), who could, and did, override clinical protocols or partograph guidance. Providers described using multiple different guidelines to guide decision‐making, without a clear consensus among respondents on which clinical protocols to use. Staff at one tertiary hospital (Hospital 1) described receiving a high number of transferred patients in advanced labor with complex medical needs, meaning intrapartum interventions were often unavoidable. Despite these challenges, providers believed that a labor monitoring tool (partograph or LCG) was beneficial as it encouraged critical thinking, served as an effective handover tool, and could be used to prompt early referral or transfer.

### 3.4 Delivery of Woman‐Centered, Respectful Care

All providers supported the practice of woman‐centered, respectful care and believed that communicating well with a woman during labor and birth was a priority. However, many felt unable to provide this care due to high patient to staff ratios, lack of privacy in shared labor room spaces, and language barriers. Doctors were responsible for obtaining informed consent and conducting patient counseling and education; however, most believed nurses could be trained to do this effectively.

### 3.5 Labor Monitoring Education and Training

All hospitals conducted regular education and training sessions for providers, but the content, delivery, and timing of these sessions varied. The three tertiary hospitals held weekly education sessions for postgraduate doctors only, while the fourth offered frequent in‐service training and upskilling for labor room nurses. Teaching sessions delivered by a consultant were comprised of didactic lectures from a broad obstetrics curriculum (not just intrapartum monitoring and management), despite providers believing this to be the least effective mode of training. Attendance was challenging as providers were unable to leave the labor room for extended amounts of time, and there was frequent turnover of staff as postgraduate doctors rotated every 3 months.

### 3.6 Audit and Feedback Procedures

All hospitals conducted regular mortality and morbidity audit meetings, which were mandatory for faculty consultants to attend. Postgraduate doctors only attended audit meetings at two hospitals, though this was not compulsory, and they were not expected to participate in discussions. Most providers believed that audit meetings could have a positive impact on partograph completion and use.

## 4 The LCG Strategy

**FIGURE 2:**
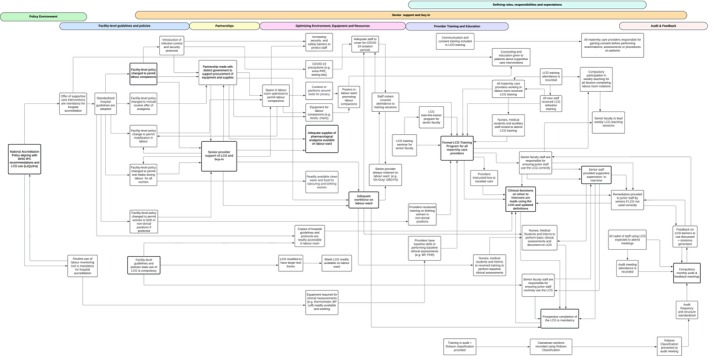
The Labour Care Guide (LCG) strategy and enabling practice environment. [Colour figure can be viewed at wileyonlinelibrary.com]

The LCG strategy comprised two key components which emerged from the final iteration of our program theory: (1) an evidence‐based LCG provider training program; and (2) regular audit and feedback cycles. However, these cannot be implemented in isolation—both rely on an enabling practice environment to support and sustain it. Using our refined program theory, we identified six key areas within the practice environment that are foundational to LCG implementation and must be addressed simultaneously: (1) a national policy framework; (2) facility‐level guidelines; (3) fostering external partnerships; (4) facility‐level senior support; (5) defining provider roles and expectations; and (6) optimizing existing equipment supplies and resources. The interaction between the LCG strategy and the practice environment in fostering behavior change is shown in Figure 2.

### 4.1 LCG Training and Education

**FIGURE 3:**
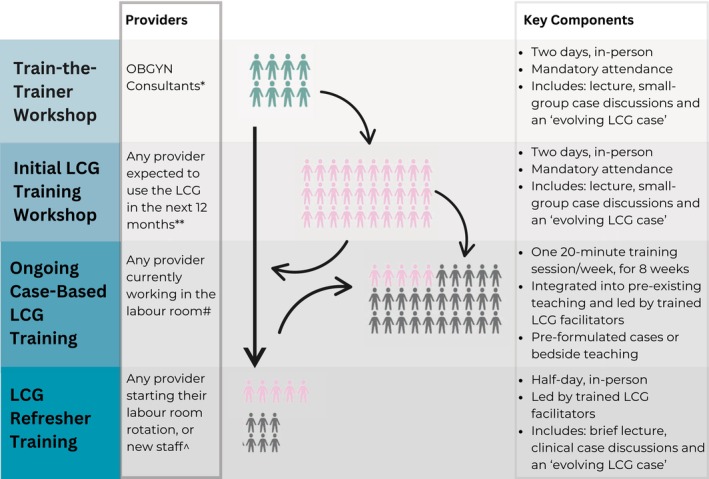
Labour Care Guide (LCG) Training and Education Program. *Any OBGYN Consultant(s) nominated to lead LCG training activities at their facility; **Attending cadre will be facility‐specific, depending on who is expected to routinely use the LCG (i.e., may include consultants, postgraduate doctors, and/or nurse‐midwives); ^#^Providers who have completed the “Initial LCG training workshop” and are currently on‐shift in the labor room (i.e., postgraduate doctors completing their 3‐month rotation); ^^^Providers who have completed the “Initial LCG training workshop” > 3 months ago, before starting their labor room rotation and/or any new staff member who will be expected to routinely use the LCG. [Colour figure can be viewed at wileyonlinelibrary.com]

The LCG training program structure is detailed in Figure 3, and the training materials we developed are in File S4. In brief, the program centers on “low dose, high frequency” training principles where content is delivered in the labor room during short, simulation‐based activities which are repeated and reinforced over time by trained facilitators [23]. This approach maximizes training exposure for providers who rotate frequently, requires no additional resources, and builds on the supportive supervision that senior staff already provide in the labor room. Importantly, the structure and content of LCG training is flexible and can be adapted to meet provider's needs. For example, ongoing LCG training sessions can be conducted at the bedside, using real‐time clinical information to discuss the complexities and nuances of decision‐making which are difficult to capture in a lecture.

### 4.2 Audit and Feedback

Monthly audit meetings are to be led by consultants and attended by labor ward staff, regardless of cadre. Recommended content includes monthly CS data using Robson Classification (overall, group 1 and group 3), and perinatal mortality and morbidity figures. These meetings should include structured, constructive discussions on how to improve overall performance, promote LCG use, and generate solutions to facility‐specific intrapartum care issues, which should be documented and re‐visited at subsequent meetings.

## 5 Discussion

This study developed a co‐designed LCG strategy to optimize and enhance implementation of the LCG in four public maternity hospitals in Karnataka, India. By applying theoretical frameworks (COM‐B/TDF) to mixed‐methods findings, we identified 142 barriers and facilitators to partograph use and labor monitoring and management practices. We used these findings to develop a priori understanding of factors that may impact LCG use and implementation in our study hospitals and used these findings to co‐design the LCG strategy. Local stakeholder engagement throughout this project ensured context sensitivity and implementation readiness. The LCG strategy we produced includes two main components: (1) an evidence‐based LCG training and education program and (2) audit and feedback cycles. Our findings emphasize that an enabling practice environment is foundational to this strategy being effective. The promising effects of this intervention in a subsequent randomized trial attest to the robustness of this approach to LCG strategy development [18].

There has been increasing recognition that partograph effectiveness is highly dependent on its implementation context and environment, rather than the design of the tool itself [9, 11, 12]. Yet evidence‐based partograph implementation strategies have been lacking, which may account for the disconnect between the partograph's effects in controlled research studies versus observations of real‐world use [9, 13]. Bedwell et al.'s realist review highlights key factors such as partograph acceptability, health system supports, adequate human resources, and provider competence as key to a “partograph enabling” environment [12]. Our findings support this, adding the importance of overarching policy frameworks, supportive and consistent facility‐level guidelines, collaborative partnerships, defining provider roles and expectations, and senior staff support as foundational to any LCG implementation efforts.

The importance of training providers in partograph use and labor monitoring and management is widely recognized [9, 12], and was repeatedly emphasized while the LCG was being developed [24]. There is, however, limited evidence on how best to train providers to use the partograph, let alone the LCG, to improve intrapartum care. A systematic review by Bluestone et al. [25] found that didactic techniques—often used in partograph education—have little to no impact on learning; rather, repetitive and short interventions are superior [25]. As part of this formative study, we developed the first evidence‐informed LCG training program, with resource‐limited settings specifically in mind. To be effective, this training must occur within a broader framework of frequent evaluation and examination of performance indicators using audit and feedback [23]. In the LCG strategy, this is achieved through monthly audit meetings attended by all labor ward staff, providing a forum to troubleshoot barriers to LCG use. CS rates, particularly those performed for women in Robson Classification group 1 and 3, were evaluated during our cluster‐randomized trial [18].

There are two limitations to our study. First, as the LCG is a novel tool, we had little direct evidence of its use and implementation in clinical practice to draw from. Thus, we used a theoretical approach to design the LCG strategy, informed by evidence on partograph use more broadly. Secondly, this study did not include the perspectives of pregnant women, and so there may be additional implementation influences that have not been considered and can be explored in future research.

## 6 Conclusion

Improving the rates of preventable maternal and intrapartum deaths, avoiding unnecessary birth interventions, and improving women's experiences of care must begin with improving the quality of labor monitoring and management. The introduction of WHO's “next generation” partograph, the LCG, provides an opportunity to do better from the start and to focus on evidence‐based implementation strategies which consider context and facility fit. We present the first evidence‐based, co‐designed LCG implementation strategy which can be used to support global LCG dissemination and uptake.

## Ethics Statement

The study protocol was approved by the Alfred Hospital Human Ethics Committee (737/20), the Institutional Ethics Committee of the KLE Academy of Higher Education and Research, Belagavi, Karnataka, India (KAHER/EC/2020‐21/D‐281120003); the Institutional Ethics Committee of J J M Medical College, Davanagere, Karnataka (JJMMC/IEC‐ 136/2020); the Institutional Ethics Committee of Vijayanagar Institute of Medical Sciences (VIMS), Ballari, Karnataka (VIMS/STD/SVN IEC/20/2020‐2021), the Institutional Ethics Committee, Gadag Institute of Medical Sciences, Gadag, Karnataka (GIMS/IEC/01/2020‐21), the State Ethics Committee, Department of Health and Family Welfare, Government of Karnataka (DD(MH)/71/2020‐21); and the Health Ministry's Screening Committee, Indian Council of Medical Research (2020‐10127).

## Conflicts of Interest

J.P.V., Y.P., V.P., F.A., and C.S.E.H. participated in technical consultations coordinated by the World Health Organization, within which the Labour Care Guide was developed. J.P.V., Y.P., V.P., F.A., and C.S.E.H. have participated in primary research that contributed to the development of the Labour Care Guide, which was financially supported by the World Health Organization. The other authors declare no conflicts of interest.

## Supporting information


**File S1:** birt70004‐sup‐0001‐SupplementaryFile1.pdf.

**File S2:** birt70004‐sup‐0002‐SupplementaryFile2.docx.

**File S3:** birt70004‐sup‐0003‐SupplementaryFile3.docx.

**File S4:** birt70004‐sup‐0004‐SupplementaryFile4.docx.
