# Supplementary material for: Developing a Co‐Designed Strategy to Improve Labor Monitoring and Management in India Using the World Health Organization Labour Care Guide: A Mixed‐Methods Formative Study
Source: Birth. 2025 Aug 13;53(1):120–8. doi: 10.1111/birt.70004 (PMC12894475; doi:10.1111/birt.70004)
Supplement: Supplementary file 1 — File S1: birt70004‐sup‐0001‐SupplementaryFile1.pdf. [file BIRT-53-120-s001.pdf]

Facility Assessment form

The aim of the Facility Assessment is to understand and describe the context in which the Labour Care Guide (LCG) implementation strategy will be carried out, and also inform the refinement and operationalisation of that strategy.

The **objectives** are:

1. Conduct a detailed assessment of a hospital's current services (including capacity, staffing, investigations, referral processes) and education and training practices
2. Determine the hospital's current clinical guidelines and protocols on labour and childbirth care
3. Identify any potential challenges to the trial implementation strategy and data collection

How to conduct this facility assessment:

- XXXX Research team to conduct an in-person visit to the hospital, including walkthrough of obstetric admissions and delivery areas; checking of equipment, supplies and medicines; inspection of 5-10 medical records. It also requires inspection of written hospital protocols, policies or guidelines.
- Meet with the Hospital Lead Investigator for this project and complete the below questions.

Table of Contents

1. Identifying information ... 3
2. Facility Services ... 4
3. Medicines, equipment, and supplies ... 7
4. Human Resources ... 9
5. Education, training, and clinical protocols ... 12
7. Referral Processes ... 16
8. Records and data management systems ... 17
9. Robson Classification ... 19
10. Quality of care audit processes ... 20
11. Physical resources and infrastructure ... 22
12. Supportive Care Interventions ... 24
13. Policies on Caesarean Section ... 27
14. Financial costs for childbirth ... 28

1. Identifying information

**1.1.** Name of hospital site	
**1.2.** Date this assessment is being completed:	
**1.3.** Type of facility (circle the right answer):	◻ Public<br>◻ Private not-for-profit hospital / NGO / Faith-based<br>◻ Private for-profit hospital<br>◻ Other / mixed ownership
**1.4.** Number of births in 2020 (1 Jan 2020 to 31 Dec 2020)	
**1.5.** Number of Caesarean section births in 2020 (1 Jan 2020 to 31 Dec 2020)	
**1.6.** Number of live births in 2020 (1 Jan 2020 to 31 Dec 2020)	
**1.7.** Number of stillbirths (from 28 weeks onwards) in 2020 (1 Jan 2020 to 31 Dec 2020)	
**1.8.** Number of maternal deaths in 2020 (1 Jan 2020 to 31 Dec 2020)	
**1.9.** Is this a university / teaching facility?	◻ Yes<br>◻ No
**1.10.** Location	◻ Urban<br>◻ Peri-urban<br>◻ Rural
**1.11.** Facility Level	◻ Primary<br>◻ Secondary<br>◻ Tertiary<br>◻ Other, please specify: ______________
**1.12.** Is this a Maternity exclusive facility?	◻ Yes<br>◻ No

2. Facility Services

2.1. Please circle to indicate which of the following services are available at this hospital.

1. Antenatal care (ANC) outpatient clinic	Y	N
2. Antenatal inpatient admission services	Y	N
a. Number of beds for antenatal admissions		
3. Laboratory diagnostic services, including any rapid diagnostic testing	Y	N
4. Inpatient labour & delivery ward	Y	N
a. Number of beds for labour admissions (first stage of labour)		
b. Number of beds in delivery area (second stage of labour)		
5. Surgical theatre (for obstetric use)	Y	N
a. How many functioning surgical theatres are there (for obstetric use)		
6. Postpartum ward	Y	N
a. Number of beds for postpartum women		
7. Postpartum care (women) outpatient clinic	Y	N
8. Postnatal care (newborns) outpatient clinic	Y	N
9. Adult intensive care unit (for obstetric use)	Y	N
a. How many intensive care unit beds are there (for obstetric use)		
10. Adult high dependency unit / beds (other than adult ICU)	Y	N
a. How many high dependency unit beds are there (for obstetric use)		
11. Newborn intensive care unit (NICU)	Y	N
a. How many NICU beds are there?		
12. Any other newborn care unit with incubators	Y	N
a. How many newborn care unit beds are there (not including NICU)?		
13. Blood bank	Y	N
14. Biochemical / clinical laboratory	Y	N
15. Ultrasound department	Y	N
16. Radiology department	Y	N
17. On-site pharmacy	Y	N

2.2. Comprehensive Emergency Obstetric Care Signal Functions

18. Does the facility have capacity to give IV antibiotics?	Y	N
19. Does the facility have capacity to give uterotonic drugs (i.e., IV oxytocin)?	Y	N
20. Does the facility have capacity to give IV anticonvulsants for pre-eclampsia and eclampsia (i.e., magnesium sulfate)?	Y	N
21. Does the facility have capacity to perform manual removal of the placenta?	Y	N
22. Does the facility have capacity to remove retained products (i.e., manual vacuum extraction, dilatation and curettage)?	Y	N
23. Does the facility have capacity to perform assisted vaginal delivery (i.e., vacuum extraction, forceps delivery)?	Y	N
24. Does the facility have capacity to perform basic neonatal resuscitation (i.e., with bag and mask)?	Y	N
25. Does the facility have capacity to perform caesarean section?	Y	N
26. Does the facility have capacity to perform blood transfusion?	Y	N

2.3. Which of the following tests could be **__performed for a woman today__?** Please circle Yes or No.

27. Temperature	Y	N
28. Blood pressure	Y	N
29. Oxygen saturation using pulse oximeter	Y	N
30. Fetal heart rate detection using Pinard stethoscope	Y	N
31. Fetal heart rate detection using Doppler device	Y	N
32. Urine dipstick (glucose, protein, ketone bodies)	Y	N
33. Urinalysis	Y	N
34. Haemoglobin / Haematocrit	Y	N
35. Platelets	Y	N
36. White cell count	Y	N
37. Liver Function Tests (ALP, GGT, AST, ALT, Bilirubin)	Y	N
38. Electrolytes and Renal Function Tests (Sodium, Potassium, Chloride, Bicarbonate, Urea, Creatinine)	Y	N

39. Coagulation tests (eg: RNI, PTT)	Y	N
40. Blood group and cross-matching	Y	N
41. Blood gas analysis/gasometry	Y	N
42. Blood cultures	Y	N
43. HIV testing	Y	N
44. Syphilis testing	Y	N
45. Malaria testing	Y	N

3. Medicines, equipment, and supplies

3.1. Please indicate whether the following medicines / treatments for women are **available today.** Please circle Yes or No.

1. Any injectable antibiotics	Y	N
2. Injectable ampicillin, gentamicin and/or clindamycin	Y	N
3. Antimalarial drugs	Y	N
4. Antiretroviral drugs	Y	N
5. Uterotonic drugs (oxytocin)	Y	N
6. Parenteral magnesium sulphate	Y	N
7. Antihypertensive agents	Y	N
8. Corticosteroids for induction of fetal lung maturation	Y	N
9. Intravenous fluids	Y	N
10. Blood products	Y	N
11. Injectable local anaesthetic drugs	Y	N
12. Fentanyl	Y	N
13. Diamorphine	Y	N
14. Pethidine	Y	N
15. Umbilical chlorhexidine	Y	N
16. Uninterrupted oxygen or CPAP (for mother)	Y	N
17. Mechanical ventilation	Y	N

3.2. Please indicate whether the following medicines / treatments for neonates are **available today.** Please circle Yes or No.

18. Injectable antibiotics for neonates	Y	N
19. Vitamin K	Y	N
20. Intravenous fluids	Y	N
21. Blood products	Y	N
22. Suction device	Y	N
23. Infant weighing scale	Y	N
24. Neonatal resuscitation with bag and mask	Y	N
25. Oxygen for newborn use	Y	N
26. Intubation	Y	N
27. Nasal CPAP	Y	N
28. Mechanical ventilation	Y	N
29. Phototherapy	Y	N
30. Vaccines against tuberculosis, hepatitis B and/or poliomyelitis	Y	N

3.3. Are the following equipment and supplies available and functioning in the **labour and delivery ward today**? Please circle Yes or No.

31. Water and soap for staff hand hygiene	Y	N
32. Hand sanitizer for staff	Y	N
33. Personal protective equipment (PPE) for health workers (i.e. masks, gowns, gloves)	Y	N
34. Clean cloth to wrap the mother	Y	N
35. Clean towels to dry and wrap the baby after birth	Y	N
36. Single use/sterile scissors or blade to cut the cord	Y	N
37. Single use/clean string/clamp to tie the cord	Y	N
38. Delivery pack	Y	N
39. Single use/sterile speculum	Y	N
40. Skin disinfectant for surgery	Y	N
41. Disposable gloves, aprons	Y	N
42. Sterile gloves	Y	N
43. Puncture proof boxes for sharps	Y	N
44. Colour coded bins for different waste products	Y	N
45. Clocks/watches	Y	N
46. Thermometer	Y	N
47. Blood pressure apparatus	Y	N
48. Intravenous infusion kits	Y	N
49. Refrigerator	Y	N
50. Well-stocked adult resuscitation trolley	Y	N
51. Well-stocked newborn resuscitation trolley	Y	N
52. Electronic fetal monitoring	Y	N
53. Ultrasound for use on labour ward	Y	N

4. Human Resources

4.1. Please describe the different healthcare provider cadres **who provide *any* sort of care to women during labour and childbirth**.

**Name of Cadre**	**Type of degree** (Certificate/Bachelor/ Masters etc)	**Place of training** (University/non-university)	**Duration of Training** (in months and years)	**Months of training specifically of labour and delivery**	**Does this cadre usually complete the partograph?**
E.g.: Midwife	*Bachelor's Degree*	*College*	*2 years*	*2 years*	*No*
E.g.: Postgraduate Doctor	*MBBS*	*University*	*6 years*	*3 months during internship*	*Yes*

4.2. Please indicate below **which cadres are employed by the hospital for the labour and delivery ward alone. Please include all staff on the payroll**. Please do not count the staff only working on ANC or PNC. If staff works part-time in the maternity, try to summarise to have full-time-equivalents.

STAFFING – LABOUR AND DELIVERY WARD ONLY		
	**Staff category**	**Number employed.** (Full-time equivalent)
1.	Consultant Obstetricians	
2.	Postgraduate doctors (i.e. residents) working in obstetrics	
3.	Interns working in obstetrics	
4.	Consultant Anaesthesiologists	
5.	Anaesthetists (nurse / paramedics)	
6.	Consultant Neonatologists	
7.	Consultant Paediatricians	
8.	Nurses working in labour ward	
9.	Midwives (exclusively trained in midwifery)	
10	Delivery assistants / auxiliary nursing staff	
11	Cleaners / other auxiliary non-nursing staff	

4.3. Please indicate below how many staff are **physically <u>available today</u> in the hospital for each shift, for labour and delivery ward alone.**

STAFFING ON SHIFTS IN LABOUR AND DELIVERY				
	**Staff category**	**DAY SHIFT** Number at the hospital (Full-time equivalent)	**NIGHT SHIFT – AT HOSPITAL** Number at the hospital (Full-time equivalent)	**NIGHT SHIFT – ON CALL** Number at home, but on call for the hospital
1.	Consultant Obstetricians			
2.	Postgraduate doctors (i.e. residents) working in obstetrics			
3.	Interns working in obstetrics			
4.	Consultant Anaesthesiologists			
5.	Anaesthetists (nurse / paramedics)			
6.	Consultant Neonatologists			
7.	Consultant Paediatricians			
8.	Nurses working in labour ward			
9.	Midwives (exclusively trained on midwifery)			
10	Delivery assistants / auxiliary nursing staff			
11	Cleaners / other auxiliary non-nursing staff			

4.4. Please briefly explain the on-call system:

- Who performs a CS during the night?
- Is the doctor to perform a CS in the hospital even at night, or is s/he on-call at home?
- Are there other resource-persons one can call in if needed?

5. Education, training, and clinical protocols

5.1. The following questions only apply to **any labour ward staff who completes a partograph** in the care of a labouring woman (e.g.: Consultant Obstetricians or Postgraduate Doctors).

1.	Does this facility conduct a **labour ward orientation** for new labour ward staff?	Y	N
2.	If yes, how often is a labour ward orientation performed each year at this facility?		
3.	Does this facility provide any **refresher training** to postgraduate doctors who rotate into labour ward?	Y	N
4.	If yes, please briefly describe the frequency and training topics:		
5.	Does the facility conduct **any regular obstetric clinical skills training or education sessions**?	Y	N
6.	If yes, please briefly describe the frequency and program contents of these trainings:		
7.	Does the facility conduct any **communication skills training** or education sessions?	Y	N
8.	If yes, please briefly describe the frequency and training contents:		
9.	Does the facility provide any **online obstetrics education or training sessions?**	Y	N
10.	If yes, please briefly describe the frequency and training contents:		
11.	Does the facility provide **any simulation or role-play-based training or education**?	Y	N
12.	If yes, please briefly describe the frequency and training contents:		

13.	Are there any mandatory **training or education modules required** to be completed before working on the labour ward? (e.g., ALSO, FSEP)?	Y	N
14.	If yes, please list here those along with their providers (e.g., local hospital, state or training college etc):		
15.	How do postgraduate doctors or OBGYN Consultants access hospital clinical guidelines and protocols? (*e.g. physical copies of clinical guidelines are printed out and given to postgraduates at start of their rotation in labour ward)*<br>Please briefly describe:		
16.	Is attendance to **clinical training and education activities mandatory** at this site?	Y	N
17.	If yes, please briefly describe how these activities are delivered to ensure all staff on rotating rosters are able to attend.		
18.	Please describe any other continuous or professional education activities ongoing in this hospital, that have not been described above.		

Protocols and guidelines for managing clinical care during labour and childbirth.

6.1. Please list the protocols available and in use in this hospital and ask about the source of guidelines (national standard guidelines e.g., by Ministry of Health, or professional organisation) and if the guideline is physically available (e.g., in a folder or displayed at the wall).

	**List of Clinical Practices**	**We have a clinical protocol for this practice.**	**Write the main Protocol you use for this practice** Please list the main protocol or guideline used (e.g. RCOG guideline, LaQshya guideline, etc)	**Is the protocol on physical display on the labour ward (eg: poster)?**
1.	Partograph use	Yes / No		
2.	Fetal Monitoring (Cartography, Intermittent Auscultation)	Yes / No		
3.	Active Management of Third Stage of labour	Yes / No		
4.	Pre-term labour	Yes / No		
5.	Indications for Induction of labour	Yes / No		
6.	Methods of Induction of labour	Yes / No		
7.	Augmentation of labour	Yes / No		
8.	Management of obstructed labour	Yes / No		
9.	Trial of vaginal birth after a caesarean section	Yes / No		
10.	Medical Documentation of Labour Care	Yes / No		
11.	Consenting the Labouring Woman	Yes / No		
12.	Analgesia in Labour	Yes / No		
13.	Routine clinical assessment of a woman in active labour (e.g. detailing indications for a vaginal examination and frequency)	Yes / No		
14.	Non-pharmacological methods of pain management in labour	Yes / No		

6.2. In the protocols and guidelines in use at this hospital, are the following interventions recommended for use in labour care practices? (tick all that apply):

- □ Offer of a labour and birth companion of choice to all women.
- □ Offer of pharmacological analgesia (including parenteral opioids or epidural)
- □ Offer of non-pharmaocological analgesia (such as massage, breathing techniques)
- □ Care providers must gain consent from all patients for any invasive procedures.
- □ Routine vaginal cleansing with chlorhexidine
- □ Encouragement of oral fluid and food intake during labour for low-risk women
- □ Encouragement of mobility and upright positioning for low-risk women
- □ The use of early amniotomy with oxytocin for augmentation for prevention of delay in labour
- □ Routine use of episiotomy
- □ Application of manual fundal pressure to facilitate childbirth.
- □ Use of uterotonics for prevention of PPH.
- □ Delayed umbilical cord clamping (for low-risk deliveries)
- □ Routine perineal/pubic shaving prior to vaginal birth.
- □ Fetal auscultation using a Doppler or Pinard to assess fetal well-being on labour admission.
- □ Use of Partograph when woman has reached 5cm dilated and is experiencing regular painful uterine contractions.

7. Referral Processes

1. Of all women giving birth in this hospital, what proportion (%) arrive following referral or transfer from another health facility? *(if you do not have an exact figure, please estimate)*	______%	
2. Does this health facility have a functional ambulance or other vehicle for emergency transportation for clients that is stationed at this facility or operates from this facility?	Y	N
3. Is fuel for the ambulance or other emergency vehicle available today?	Y	N
4. Is emergency transport free for patients?	Y	N
5. Of all women who attend this hospital to give birth, what proportion (%) need to be transferred to another, higher-level hospital?	______%	
6. How far is your hospital from the nearest higher-level hospital (if you needed to transfer women to a higher level of care)?	___________________(km)	

8. Records and data management systems

There are typically two different places for documenting information about births:

a) facility-level register or log book of births occurring in labour ward; and

b) case notes/patients medical record.

8.1. Please describe the **facility-level register or log book** collating key obstetric variables at the facility-level.

**Question**	**Example Answer:**	**Your Answer:**
Is it paper or electronic?	*Paper based logbook*	
Where is it located?	*At nursing station in labour ward*	
Who is responsible for completing it?	*The nurse who attended the birth*	
When do they complete it?	*Usually within 6 hours after birth*	
What maternal information does it capture?<br>(list all variables)	• *Woman's name,*<br>• *Medical ID number*<br>• *Maternal age*<br>• *Maternal parity*<br>• *Date of child birth,*<br>• *Mode of birth*	
What newborn information does it capture?	• *Gestational age at birth*<br>• *Vital Signs*<br>• *Birthweight*<br>• *Apgar at 5 min*<br>• *NICU admission yes or no*	
How often is this register summarised?	*Eg: used to prepare monthly report, in first week of the month*	

Now concerning a woman's case notes/medical records:

8.2. Please describe the medical record structure (e.g., electronic or paper), and who keeps the records (e.g., woman or provider)? Is there a standardised form?

8.3. Who is responsible for completing the **individual-level** medical records?

9. Robson Classification

Please review 5-10 medical records for women who have given birth in the past 7 days.

9.1. For each of these records, please indicate whether the Robson Classification variable is clearly recorded.

**Number**	**Parity is clearly recorded**	**Previous Caesarean section or not is clearly recorded**	**Onset of labour is clearly recorded.** (spontaneous, induced or pre-labour CS)	**Gestational age at birth Is clearly recorded?**	**Fetal presentation at birth is clearly recorded (cephalic, breech, or transverse/oblique)**	**Number of fetuses is clearly recorded (singleton, multiple)**	**Final mode of birth is clearly recorded (vaginal or CS)**
E.g.:001	***Y***	***N***	***Y***	***Y***	***Y***	***Y***	***Y***
E.g. 002	***Y***	***Y***	***Y***	***N***	***Y***	***Y***	***Y***

10. Quality of care audit processes

**1.** Does this hospital have a regular meeting to review **maternal deaths?**	◻ Yes<br>◻ No
a. If yes, how often do they meet?	◻ Weekly<br>◻ Monthly<br>◻ Every 3 months<br>◻ Every 6 months<br>◻ Once a year<br>◻ Other: ______
b. Who Chairs this meeting? (name and position)	
**2.** Does this hospital have a regular meeting to review **severe maternal morbidity events (eg: maternal near miss)?**	◻ Yes<br>◻ No
a. If yes, how often do they meet?	◻ Weekly<br>◻ Monthly<br>◻ Every 3 months<br>◻ Every 6 months<br>◻ Once a year<br>◻ Other: ______
b. Who Chairs this meeting? (name and position)	
**3.** Does this hospital have a regular meeting to review the **Caesarean section rate?**	◻ Yes<br>◻ No
a. If yes, how often do they meet?	◻ Weekly<br>◻ Monthly<br>◻ Every 3 months<br>◻ Every 6 months<br>◻ Once a year<br>◻ Other: ______
b. Who Chairs this meeting? (name and position)	
c. Please circle all the cadres which are meant to attend the regular meeting on Caesarean section rate?	◻ Consultant Obstetricians<br>◻ Postgraduate Residents working in labour ward<br>◻ Interns working in labour ward<br>◻ Nurses working in labour ward

10.1. Does this hospital conduct any clinical audits on the Caesarean Section rate? If yes, please briefly describe these activities.

Example: Yes. The Caesarean rate data is reviewed in a monthly meeting of all Obstetric Consultants, where we discuss strategies to improve the CS rate.<br><br>*Example: The CS rate for that month is communicated to staff through an email newsletter and a poster on the noticeboard.*<br><br>*Example: we do not conduct any routine audits on Caesarean section.*

10.2. Does this hospital routinely use the Robson Classification in assessing the Caesarean Section rate? If yes, please briefly describe how it is used.

Example: we do not use the Robson Classification<br>*Example: For the past year, one of our postgraduate students has analysed CS data using Robson Classification. They presented the data at a Departmental meeting. This was the first time we have used Robson.*

11. Physical resources and infrastructure

Please observe the physical space of the labour, delivery and postnatal wards. If these are in separate areas (e.g.: women in latent labour in a labour ward, women in active labour in a separate room/delivery ward, separate postnatal ward), please assess all areas according to all points below.

11.1. Inventory of physical space and resources

Please sketch a floor plan of the labour ward itself, including admissions area. Please write the number of beds per room or area.

11.2. Infrastructure

Are the following resources available and functioning in this facility **today**? Circle Yes or No.

1. Electricity from any power source during the last 24 hours	Y	N
2. Generator	Y	N
3. Incinerator	Y	N
4. Sewage system	Y	N
5. Sterilization facilities/equipment (eg: autoclaving)	Y	N
6. Disinfectants for instruments	Y	N
7. Functioning land line telephone / short-wave radio to call outside	Y	N
8. Functional ambulance available	Y	N
9. Printer	Y	N
10. Computer with internet connection	Y	N

12. Supportive Care Interventions

12.1. Labour and Birth Companions

1. At this hospital, are women giving birth permitted to have a companion present?	◻ Yes<br>◻ No<br><br>**If companions are not permitted under any circumstances, Go to Section 12.2**
2. If yes, who is allowed to act as companion of the woman? Tick all that apply.	__ Husband/male partner<br>__ Female family member<br>(eg: Mother, sister, mother-in-law)<br>__ Friend<br>__ Doula
3. At what times are a companion permitted to be present?	
a. Labour / first stage	Yes / no
b. Delivery / second stage	Yes / no
c. Immediate postpartum period (first hour or two after birth)	Yes / no
d. Postnatal ward	Yes / no
e. Day-time	Yes / no
f. Night-time	Yes / no
4. Which of the following tasks are a companion allowed to do in your hospital? Tick all that apply	___ provide emotional support to woman (eg: praise, reassurance)<br>___ fetching or providing food or drink to woman<br>___ massaging the woman<br>___ helping woman to move or walk around<br>___ providing information to woman (eg: repeating or relaying information from the health worker)<br>___ asking questions to health worker on the woman's behalf<br>___ helping soiled linens or clothes<br>___ language translation (if needed)

12.2. Pain relief

Please indicate which of the following pain relief options are available:

	Available	Routinely offered
Manual techniques, such as massage or application of warm packs	☐ Always<br>☐ Sometimes<br>☐ Never	☐ Always<br>☐ Sometimes<br>☐ Never
Relaxation techniques, including progressive muscle relaxation breathing, music, mindfulness and other techniques	☐ Always<br>☐ Sometimes<br>☐ Never	☐ Always<br>☐ Sometimes<br>☐ Never
Parenteral opioids, such as fentanyl, diamorphine and pethidine	☐ Always<br>☐ Sometimes<br>☐ Never	☐ Always<br>☐ Sometimes<br>☐ Never
Epidural analgesia	☐ Always<br>☐ Sometimes<br>☐ Never	☐ Always<br>☐ Sometimes<br>☐ Never

12.3. Oral fluids and food intake

At this hospital, are low-risk women admitted for childbirth <u>permitted</u> to take oral fluids?	☐ Always<br>☐ Sometimes<br>☐ Never
At this hospital, are low-risk women admitted for childbirth <u>encouraged</u> to take oral fluids?	☐ Always<br>☐ Sometimes<br>☐ Never
At this hospital, are low-risk women admitted for childbirth <u>allowed</u> to take food?	☐ Always<br>☐ Sometimes<br>☐ Never
At this hospital, are low-risk women admitted for childbirth <u>encouraged</u> to take food?	☐ Always<br>☐ Sometimes<br>☐ Never

12.4. Does the hospital have a written policy regarding oral fluid and food intake for women giving birth? If yes, please briefly describe the policy below.

12.5. Mobilisation during labour

At this hospital, are women admitted for childbirth <u>permitted</u> to walk around while in labour?	☐ Always<br>☐ Sometimes<br>☐ Never

At this hospital, are women admitted for childbirth encouraged to walk around while in labour?	□ Always<br>□ Sometimes<br>□ Never

Does the hospital have a written policy regarding women walking or moving around during labour? If yes, please briefly describe the policy below.

12.6. Birth position of choice

Are women encouraged to give birth in a position of their choice (eg: supine or semi-recumbent position, squatting, kneeling, and on hands and knees) If not, why not?

12.7. Measuring women's care experiences

Does the hospital use a method of routinely measuring women's experiences at birth? For example, through postpartum questionnaires or consumer surveys.

13. Policies on Caesarean Section

13.1. Please briefly explain your hospital's usual process for **obtaining informed consent** for Caesarean section:

- Which staff member usually obtains informed consent?
- When is informed consent usually obtained?
- How provides informed consent?
- Is consent documented? If yes, where and how?

13.2. **Caesarean section on maternal request only -** Please provide a brief description of what is the current hospital policy regarding caesarean section on maternal request. Please specify if no such policy exists.

13.3. **Trial of labour and vaginal birth after caesarean section -** Please provide a description of what is the current hospital policy regarding trial of labour and/or vaginal birth after caesarean section. Please specify if no such policy exists.

13.4. What is the average time taken from "Decision for Caesarean Section" to "Knife-to-Skin" for an emergency caesarean section? (surgical theatre audit data may assist with this)

13.5. According to current practice at this hospital, when is the partograph expected to be started?

14. Financial costs for childbirth

14.1. Formal cost of delivery

Please provide a brief description of the financial costs to the woman and her family for a vaginal birth and for a caesarean section at this hospital.

14.2. Informal cost of delivery

Please provide a description of any informal costs to the woman and her family (e.g. under the table payment, envelopes, informal payments to the providers)

