# Supplementary material for: Developing a Co‐Designed Strategy to Improve Labor Monitoring and Management in India Using the World Health Organization Labour Care Guide: A Mixed‐Methods Formative Study
Source: Birth. 2025 Aug 13;53(1):120–8. doi: 10.1111/birt.70004 (PMC12894475; doi:10.1111/birt.70004)
Supplement: Supplementary file 2 — File S2: birt70004‐sup‐0002‐SupplementaryFile2.docx. [file BIRT-53-120-s004.docx]

| **Survey of Maternity Care Providers** This survey is for any maternity care provider working in labour ward (Consultant OBGYN, Postgraduate Doctors, Medical Interns, Nurses, Midwives, or students) across four hospitals participating in this trial.  We would like to understand your professional background, your day-to-day clinical work, and what it is like to provide labour and childbirth care in your hospital.  **Your responses are anonymous, and you will not be identified in any way.** Your responses will not be viewed by your clinical supervisors or colleagues.  Please answer the below questions which will take 10 – 15 minutes to complete.  If there is any question you do not wish to answer, you can leave it blank and move to the next question. |
| --- |

**Please write the date you are completing this survey: _____________**

### **Section 1: Demographics**

- 1. Which hospital do you currently work in?
- XXX
- XXX
- XXX
- XXX
  1. What is your current position title (select one only)?
- Consultant Obstetrician-Gynaecologist
- Postgraduate Medical Doctor
- Medical Intern
- Medical Academic / Non-Clinical Doctor
- Nurse
- Midwife
- Nurse-Midwife
- Medical Student
- Nursing or Midwifery Student
- Other (please specify): _______________
  1. How many **years and months in total** have you worked in this role, including at different hospitals?

------- YEARS ------- MONTHS

- 1. How many years and months have you been **in your current position** **at this hospital**?

------ YEARS ------- MONTHS

- 1. What is the highest professional qualification you have obtained?
- Doctoral Degree
- Master’s Degree
- Bachelor Degree
- Professional Fellowship (e.g. FOGSI)
- Graduate Certificate or Diploma
- High School Certificate / Completion
  1. When did you last monitor a woman during labour?
- In the last month
- 1 to 6 months ago
- Over 6 months ago
- I have never monitored a woman in labour
  1. When did you last assist a woman having a vaginal birth?
- Less than 1 month
- 1 to 6 months ago
- Over 6 months ago
- I have never assisted with a vaginal birth
  1. When did you last perform or assist in a Caesarean section?
- Less than 1 month
- 1 to 6 months ago
- Over 6 months ago
- I have never performed or assisted in a Caesarean section
  1. In an average week, how many births are you involved in? (e.g. oversee the management of, actively assist the birth, care for the woman throughout labour etc)
- Less than 10 births
- Between 10 – 50 births
- Between 50 – 100 births
- Over 100 births
  1. Please tick any of the following tasks (one or more) that you usually do in your current role:
- Triage and admission to labour ward
- Measuring a woman’s vital signs (i.e. temperature, blood pressure etc)
- Providing a clean bed and linen
- Performing fetal assessment (using Pinard or fetal doppler)
- Performing an abdominal examination
- Assessing for ruptured membranes
- Performing a vaginal examination
- Writing a woman’s progress in labour on a partograph
- Helping the woman to be mobile in labour
- Providing support and encouragement to the woman
- Offering a woman analgesia
- Allowing a woman to have a labour companion
- Making decisions on what interventions to use during labour (e.g. when to use oxytocin, artificial rupture of membranes)
- Making a decision on when to perform a Caesarean section
- Making a decision on when to perform an instrumental delivery (e.g. forceps)
- Making a decision on when to perform an episiotomy
- Consenting a woman for a Caesarean section
- Supporting the woman to have a vaginal birth
- Performing (or assisting) on a Caesarean section
- Controlling visitors to labour ward

### **Section 2: Your experiences with caring for women during labour and childbirth**

Considering your current role at this hospital, to what extent do you agree or disagree with the following statements? Please tick the box that you feel applies to you.

|  | **Strongly disagree** | **Disagree** | **Neither agree nor disagree** | **Agree** | **Strongly agree** | **Unsure** |
| --- | --- | --- | --- | --- | --- | --- |
| **2.1**. I feel that the quality of care women receive during labour at my hospital needs improvement |  |  |  |  |  |  |
| **2.2.** I feel that the quality of care women receive during labour at my hospital is compromised due to staff workloads |  |  |  |  |  |  |
| **2.3.** I find it difficult to get help from my seniors when I need it (e.g., Senior Nurse or Senior Doctor) |  |  |  |  |  |  |
| **2.4.** In my work on labour ward, I feel like I am caring for too many women at one time |  |  |  |  |  |  |
| **2.5.** Women giving birth in my hospital **during the day** are able to get a Caesarean section quickly if they need it |  |  |  |  |  |  |
| **2.6.** Women giving birth in my hospital **during the night** are able to get a Caesarean section quickly if they need it |  |  |  |  |  |  |
| **2.7.** Overall, I feel I provide good-quality care to women during labour and birth |  |  |  |  |  |  |
| **2.8.** I feel I have had adequate education and training to provide good-quality labour care |  |  |  |  |  |  |
| **2.9.** Women should have a companion of their choosing (such as a family member) present during labour or birth if they want |  |  |  |  |  |  |

### **Section 3. Current labour care practice**

Considering your current role at this hospital, to what extent do you agree or disagree with the following statements? Please tick the box that you feel applies to you.

|  | **Strongly disagree** | **Disagree** | **Neither agree nor disagree** | **Agree** | **Strongly agree** | **Unsure** |
| --- | --- | --- | --- | --- | --- | --- |
| **3.1**. I am able to give women in my care one-to-one support and encouragement during labour |  |  |  |  |  |  |
| **3.2.** I find it difficult to find the time to communicate effectively with women who give birth under my care |  |  |  |  |  |  |
| **3.3.** Women under my care receive vaginal examinations in private (i.e. in such a way that no one else can see them) |  |  |  |  |  |  |
| **3.4.** I am able to maintain a labouring woman’s privacy and confidentiality in the current labour ward setting |  |  |  |  |  |  |
| **3.5.** I do not think communicating with labouring women is an important priority |  |  |  |  |  |  |

When a woman is admitted in labour, please indicate how often you perform the following interventions? Tick the box that best applies to you.

|  | **Always** | **Often** | **Sometimes** | **Occasionally** | **Never** | **Unsure** |
| --- | --- | --- | --- | --- | --- | --- |
| ***Labour companionship*** |  |  |  |  |  |  |
| **3.6.** I offer to have a companion of their choosing (such as a family member) present during labour |  |  |  |  |  |  |
| **3.7.** I offer to have a companion of their choosing (such as a family member) present during vaginal birth |  |  |  |  |  |  |
| ***Pain management during labour*** |  |  |  |  |  |  |
| **3.8.** I offer some form of pain relief to women under my care |  |  |  |  |  |  |
| **3.9**. Women under my care are able to receive an epidural if they choose |  |  |  |  |  |  |
| **3.10.** Women under my care are able to receive an opiate (such as fentanyl or diamorphine) if they choose |  |  |  |  |  |  |
| **3.11**. Women under my care are able to use relaxation techniques, such as mobility, breathing or music, if they choose |  |  |  |  |  |  |
| **3.12.** Women under my care are able to receive manual pain relief techniques (such as massage or warm packs) if they choose |  |  |  |  |  |  |

When a woman is admitted in labour, please indicate how often you perform the following interventions? Tick the box that best applies to you.

|  | **Always** | **Often** | **Sometimes** | **Occasionally** | **Never** | **Unsure** |
| --- | --- | --- | --- | --- | --- | --- |
| ***Oral fluids and diet*** |  |  |  |  |  |  |
| **3.13.** Low-risk women under my care are encouraged to drink oral fluids and eat during labour |  |  |  |  |  |  |
| ***Posture and mobility*** |  |  |  |  |  |  |
| **3.14.** Women under my care are encouraged to walk or move around during labour |  |  |  |  |  |  |
| **3.15.** Women under my care who do not have an epidural can give birth in any position of their choice (sitting, squatting, kneeling, etc) |  |  |  |  |  |  |
| **3.16**. Women under my care who have an epidural can give birth in any position of their choosing (sitting, squatting, kneeling etc) |  |  |  |  |  |  |

### **Section 4: Education and Training**

*Next, we would like to focus on any training you have received around the care of women during labour and childbirth.*

- 1. While working in this hospital, have you received any additional training or education (eg. In-service education or refresher) on **managing women in labour**?
- No, I have not received any additional training or education (**move to Q4.2)**
- Yes, I have received some form of training or education on managing women in labour.
  - 1. If yes, please briefly describe what education and training you received, and the frequency of this training (e.g. every week for two months, yearly etc):

----------------------------------------------------------------------------------------------------------------------------------------------------------------------------------------------------------------------------------------------------

- - 1. If yes, how long ago did you receive **your most recent training** **on managing women in labour**?
- <1 year
- 1-2 years
- 3-5 years
- >5 years
  - 1. At **your most recent training** **on managing women in labour**, who was this training given by? (Please tick all that apply)
       - Your peers (e.g. other doctors, midwives or nurses at your clinical level)
       - Your clinical supervisor/senior (e.g. Consultant Obstetrician, Senior Nurse)
       - Non-clinical trainers from your hospital or affiliated university
       - Trainers from the district or regional team
       - Trainers from another organisation or International Agency (e.g. UNFPA, WHO)
       - Trainers from the Ministry of Health
       - Other (please specify) ___________________________________
       - I do not know.
    2. At **your most recent training** **on managing women in labour** - what was the format of the training? Please tick all that apply.
- Workshop
- Presentation/Lecture
- Simulation (e.g. using mannequins or models)
- Role-play
- Case-based
- Online modules
- Training videos
- Self-directed learning
- Other (Please specify) ___________________________________

Considering your current role at this hospital, to what extent do you agree or disagree with the following statements? Please tick the box that you feel applies to you.

|  | **Strongly disagree** | **Disagree** | **Neither agree nor disagree** | **Agree** | **Strongly agree** | **Unsure** |
| --- | --- | --- | --- | --- | --- | --- |
| **4.2.** At my hospital, the education and training on managing labour is relevant to my clinical responsibilities |  |  |  |  |  |  |
| **4.3**. I have been adequately trained in providing care to women for a vaginal birth |  |  |  |  |  |  |
| **4.4.** I have been adequately trained in providing care to women for a Caesarean birth |  |  |  |  |  |  |
| **4.5.** I have been adequately trained in how to fill out a partograph |  |  |  |  |  |  |
| **4.6.** I have been adequately trained on how to provide supportive care to women during labour and childbirth |  |  |  |  |  |  |
| **4.7.** I am confident in my ability to clinically assess a woman in labour (e.g. doing abdominal palpation to assess fetal position, doing vaginal examination to determine cervical dilation, station, position etc) |  |  |  |  |  |  |
| **4.8.** I feel confident in my decisions to medically intervene in a women’s labour (e.g. augment labour, rupture membranes, perform episiotomy etc) |  |  |  |  |  |  |
| **4.9.** I would benefit from additional clinical training and education in managing women during labour and birth |  |  |  |  |  |  |
| **4.10.** I am regularly supervised by senior clinicians (senior nurses or doctors) at my hospital |  |  |  |  |  |  |
| **4.11.** I receive feedback from my seniors (senior nurses or doctors) on my performance |  |  |  |  |  |  |
| **4.12**. My workload prevents me from attending education and training at this hospital |  |  |  |  |  |  |

**4.13**. From the following list, which modes of education and training do you find the most effective in improving your skills in managing women in labour? Please tick a **maximum of** **three options**

- Presentation/Lecture
- Workshop
- Simulation (e.g. use of mannequins or models)
- Role-play
- Case-based learning
- Online modules
- Training videos
- Monthly meetings with colleagues
- Other (please specify):

### **Section 5: Guidelines and Protocols**

*We would now like to ask you a few questions about guidelines. By guidelines, we mean any hospital, district, national, or international recommendations you frequently refer to when managing labour and childbirth.*

**5.1.** Which clinical guidelines or protocols do **you usually refer to** when monitoring women in labour at your hospital (e.g. FOGSI guidelines, local hospital guidelines, RCOG, etc)?

- This hospital’s Clinical Guidelines
- District or State Clinical Guidelines
- Indian National Clinical Guidelines
- Clinical Guidelines from another country (such as American College of Obstetrics and Gynaecology Guidelines, Royal College of Obstetrics and Gynaecology Guidelines)
- World Health Organization Clinical Guidelines
- Other Clinical Guidelines (Please Specify):

---------------------------------------------------------------------------------------------------------------------

Considering your current role at this hospital, to what extent do you agree or disagree with the following statements? Please tick the box that you feel applies to you.

|  | **Strongly disagree** | **Disagree** | **Neither agree nor disagree** | **Agree** | **Strongly agree** | **Unsure** | **Not applicable** |
| --- | --- | --- | --- | --- | --- | --- | --- |
| **5.2**. I regularly follow clinical guidelines when I am managing women in labour |  |  |  |  |  |  |  |
| **5.3.** I find guidelines useful for my clinical practice |  |  |  |  |  |  |  |
| **5.4.** At this hospital, women in labour do not receive care as recommended in guidelines |  |  |  |  |  |  |  |
| **5.5**. I can easily access a copy of clinical guidelines for managing women in labour in my hospital |  |  |  |  |  |  |  |
| **5.6.** In general, I think that clinical guidelines are followed regularly at my hospital |  |  |  |  |  |  |  |
| **5.7.** I find it difficult to keep up with changes to guidelines |  |  |  |  |  |  |  |

### **Section 6: Using the partograph**

Please indicate your views on the following questions. Tick the box that best applies to you.

|  | **Always** | **Often** | **Sometimes** | **Occasionally** | **Never** | **Unsure** |
| --- | --- | --- | --- | --- | --- | --- |
| **6.1.** Women under my care are managed using a partograph |  |  |  |  |  |  |
| **6.2.** Women under my care have their partograph completed as it happens (i.e. during labour) |  |  |  |  |  |  |
| **6.3.** Women under my care have their partograph completed afterwards (i.e. after birth has occurred) |  |  |  |  |  |  |
| **6.4.** At my hospital, partographs are filled out incorrectly |  |  |  |  |  |  |
| **6.5.** I am encouraged by senior staff to ensure a partograph is used correctly |  |  |  |  |  |  |
| **6.6.** At my hospital, the equipment needed to use the partograph is available when needed |  |  |  |  |  |  |
| **6.7.** At my hospital, staff find it hard to find the time to fill out the partograph – either during labour, or afterwards |  |  |  |  |  |  |
| **6.8**. At my hospital, a lack of staff restricts use of the partograph |  |  |  |  |  |  |
| **6.9.** I know how to manage a woman in labour and I do not need to use a partograph |  |  |  |  |  |  |

Considering your current role at this hospital, to what extent do you agree or disagree with the following statements? Please tick the box that you feel applies to you.

|  | **Strongly disagree** | **Disagree** | **Neither agree nor disagree** | **Agree** | **Strongly agree** | **Unsure** | **Not applicable** |
| --- | --- | --- | --- | --- | --- | --- | --- |
| **6.10.** I know how to complete a partograph correctly. |  |  |  |  |  |  |  |
| **6.11.** I feel I am well-trained in using a partograph correctly. |  |  |  |  |  |  |  |
| **6.12.** I have been taught how to use a partograph correctly. |  |  |  |  |  |  |  |
| **6.13**. My supervisors care about whether the partograph has been completed correctly. |  |  |  |  |  |  |  |
| **6.14.** I find my hospital’s partograph easy to understand. |  |  |  |  |  |  |  |
| **6.15.** I find my hospital’s partograph easy to use. |  |  |  |  |  |  |  |
| **6.16.** I think that using a partograph improves women’s health outcomes. |  |  |  |  |  |  |  |
| **6.17.** I think using a partograph improves the quality of care women receive. |  |  |  |  |  |  |  |
| **6.18.** I think using a partograph improves women’s satisfaction with childbirth |  |  |  |  |  |  |  |
| **6.19.** Completing a partograph is not a good use of staff time. |  |  |  |  |  |  |  |
| **6.20.** I feel supported by senior staff to use a partograph correctly. |  |  |  |  |  |  |  |

### **Section 7: Equipment and supplies**

Considering your current role at this hospital, to what extent do you agree or disagree with the following statements? Please tick the box that you feel applies to you.

|  | **Always** | **Often** | **Sometimes** | **Occasionally** | **Never** | **Unsure** |
| --- | --- | --- | --- | --- | --- | --- |
|  |  |  |  |  |  |  |
| **7.1.** I have access to the supplies I need to care for women during labour and birth |  |  |  |  |  |  |
| **7.2.** There is an adequate supply of pain medication (e.g. opiates) for women during labour and birth |  |  |  |  |  |  |
| **7.3.** Women can labour and give birth in private (i.e. in a private room, or room sectioned off with curtains so no-one can see) |  |  |  |  |  |  |
| **7.4**. I can obtain a blank paper partograph when I need one |  |  |  |  |  |  |
| **7.5.** There is enough space in a labour ward area for a labour companion to be present |  |  |  |  |  |  |
| **7.6.** I can obtain a blood pressure cuff when I need one |  |  |  |  |  |  |
| **7.7.** I can obtain a stethoscope when I need one |  |  |  |  |  |  |
| **7.8.** I can obtain a thermometer when I need one |  |  |  |  |  |  |
| **7.9.** I can obtain a fetoscope or Pinard stethoscope when I need one |  |  |  |  |  |  |

- 1. . Are there any other equipment or supply issues that restrict you from providing quality labour care in your current role at this hospital? Please briefly describe below.

**Section 8: Improving management of labour and childbirth.**

Finally, we would like to ask some questions about improving labour and childbirth in your hospital specifically.

Considering your current role at this hospital, to what extent do you agree or disagree with the following statements? Please tick the box that you feel applies to you.

|  | **Strongly disagree** | **Disagree** | **Neither agree nor disagree** | **Agree** | **Strongly agree** | **Unsure/no opinion** |
| --- | --- | --- | --- | --- | --- | --- |
| **8.1** The Caesarean section rate at our hospital is too high |  |  |  |  |  |  |
| **8.2** The Caesarean section rate at our hospital is too low |  |  |  |  |  |  |
| **8.3** The Caesarean section rate at our hospital is about right |  |  |  |  |  |  |

- 1. Are any of the following strategies in place at your hospital to assist you in providing quality labour and childbirth care? Please tick all that apply.
  - Decision aids (e.g., clinical management algorithms)
  - Posters on how to manage labour which are clearly displayed in labour ward.
  - Verbal feedback from senior clinical staff
  - Clinical case debriefs with senior clinical staff and/or peers
  - Regular clinical audits
  - Guidelines and protocols on display
  - Formal peer support from my colleagues (e.g., mentorship)
  - I don’t know.
  - None of these strategies are in place.
  - Other (please specify): __________________

How effective do you think each of the following strategies might be in improving managing women in labour at your hospital?

|  | **Not at all effective** | **Slightly effective** | **Moderately effective** | **Very effective** | **Extremely effective** | **Unsure** |
| --- | --- | --- | --- | --- | --- | --- |
| **8.6**. Lectures or presentations |  |  |  |  |  |  |
| **8.7.** Training workshops |  |  |  |  |  |  |
| **8.8.** Decision aids (e.g., management algorithms) |  |  |  |  |  |  |
| **8.9.** Posters or printed protocols/guidelines |  |  |  |  |  |  |
| **8.10.** Verbal feedback from senior clinical staff |  |  |  |  |  |  |
| **8.11.** Regular clinical audits |  |  |  |  |  |  |
| **8.12.** Clinical case debriefs with senior clinical staff and/or peers |  |  |  |  |  |  |
| **8.13.** Peer support from my colleagues |  |  |  |  |  |  |
| **8.14**. Improving access to clinical equipment |  |  |  |  |  |  |
| **8.15.** Improving access to supplies |  |  |  |  |  |  |
| **8.16.** Improving access to medicines |  |  |  |  |  |  |

### **Section 9: COVID-19**

- 1. What impact has COVID-19 had on your practice? Tick any that apply to you.
- The number of women attending hospital to give birth has increased.
- The number of women attending hospital to give birth has reduced.
- We have more supplies than before the outbreak.
- We have less supplies than before the outbreak.
- We have more staff than before the outbreak.
- We have less staff than before the outbreak.
- I am working more hours than before the outbreak.
- I am working less hours than before the outbreak.
- There are new policies and guidelines to follow.
- All labouring women are still allowed to have one birth companion of their choosing.
- All labouring women are not permitted to have a birth companion.

### **Section 10: Thank You**

Thank you for taking the time to complete this survey. Your contribution is appreciated.

If there is anything further you would like to share about anything covered in this survey, please feel free to do so below:
