# Supplementary material for: Developing a Co‐Designed Strategy to Improve Labor Monitoring and Management in India Using the World Health Organization Labour Care Guide: A Mixed‐Methods Formative Study
Source: Birth. 2025 Aug 13;53(1):120–8. doi: 10.1111/birt.70004 (PMC12894475; doi:10.1111/birt.70004)
Supplement: Supplementary file 3 — File S3: birt70004‐sup‐0003‐SupplementaryFile3.docx. [file BIRT-53-120-s003.docx]

| **Target Behaviour** | **COM-B** | **TDF** | **List of factors affecting the target behaviour** | **B** | **F** | **M** |
| --- | --- | --- | --- | --- | --- | --- |
| **Routinely offering supportive care** | C | Phys | Providers felt they already had the required skills to offer women supportive care |  |  |  |
|  | C | Know | Providers felt that in-depth knowledge was not required to offer women supportive care |  |  |  |
|  | C | Beh Reg | Using the supportive care section on the LCG is an effective prompt or reminder |  |  |  |
|  | C | Beh Reg | Government policy (LaQysha) mandates routine offer of supportive care interventions, and this accreditation provided financial resources to hospitals |  |  |  |
|  | O | Env | Posters promoting supportive care interventions are displayed in the labour room |  |  |  |
|  | O | Env | Current hospital policies do not permit labour companionship |  |  |  |
|  | O | Env | There is inadequate space in labour ward to accommodate a labour companion |  |  |  |
|  | O | Env | There are not enough supplies to accommodate labour companions (e.g. stools or chairs) |  |  |  |
|  | O | Env | There is inadequate safety barricades and security staff to limit number of labour companions |  |  |  |
|  | O | Env | Current hospital guidelines do not uniformly recommend routine offer of any type of analgesia (pharmacological or non-pharmacological) |  |  |  |
|  | O | Env | There are inadequate and inconsistent supplies of all forms of pharmacological analgesia |  |  |  |
|  | O | Env | Use of epidural analgesia is reserved only for operative births |  |  |  |
|  | O | Env | Non-pharmacological pain relief options (e.g. heat packs) are available on labour wards |  |  |  |
|  | O | Env | All hospitals had clinical guidelines on avoiding dehydration in labour |  |  |  |
|  | O | Env | All women had access to clean water in the hospital facilities |  |  |  |
|  | O | Env | Hospitals did not provide meals to women during labour |  |  |  |
|  | O | Env | Oral intake was only permitted for ‘low risk’ women |  |  |  |
|  | O | Env | Mobilisation in labour for ‘low risk’ women was permitted |  |  |  |
|  | O | Env | In practice, mobilisation in labour was limited by lack of analgesia (i.e. women were in pain and unable to mobilise easily) and lack of space in labour ward in which to mobilise |  |  |  |
|  | O | Env | Insufficient staff time to provide supportive care to patients |  |  |  |
|  | M | Bel Cap | Providers do not feel confident to birth women in non-dorsal positions |  |  |  |
|  | M | Bel Con | Perceived infection control risks (COVID) with implementation of labour companionship |  |  |  |
|  | M | Bel Con | Perceived safety risks to labour ward staff with implementation of labour companionship |  |  |  |
|  | M | Bel Con | Perceived infection risk to patients with implementation of labour companionship |  |  |  |
|  | M | Bel Con | Perceived risk to patient privacy with implementation of labour companionship |  |  |  |
|  | M | Bel Con | Concerns that labour companions would interfere with patient care |  |  |  |
|  | M | Bel Con | Aspiration risk concerns if oral intake is offered to all women |  |  |  |
|  | M | Bel Con | Concerns that labour companionship policies would lead to increased COVID infections amongst staff, leading to staff shortages |  |  |  |
|  | M | Goals | Develop partnerships with district government officials to assist with procurement of supplies (e.g. drugs for analgesia) |  |  |  |
|  | M | Goals | Commitment from hospitals to gain LaQysha accreditation and incentives through offering supportive care measures |  |  |  |
|  | M | Opt | Provider beliefs that with adequate drug supplies, pharmacological analgesia can be routinely offered |  |  |  |
|  | M | Id | Different cadres of providers were unclear as to their roles and responsibilities in offering supportive care |  |  |  |
|  | M | Id | Providers believed offering women pharmacological analgesia was the role of the doctor |  |  |  |
|  | M | Id | Providers believed encouragement of mobilisation in labour was the role of a nurse/nurse-midwife |  |  |  |
|  | M | Id | Certain cadres were not in favour of routinely offering all supportive care interventions |  |  |  |
|  | M | Em | High provider satisfaction with using the supportive care section of the LCG |  |  |  |

| **Consistent, prospective partograph use** | C | Phys | Not all providers have the skills required to perform clinical measurements or examinations required for partograph completion (e.g. fetal heart rate or cervical dilation assessment) |  |  |  |
| --- | --- | --- | --- | --- | --- | --- |
|  | C | Phys | Providers assume that all labour ward staff already have adequate skills in labour monitoring, acquired during pre-service training (eg: medical school) |  |  |  |
|  | C | Know | Providers have inconsistent knowledge of WHO intrapartum care recommendations and LCG use |  |  |  |
|  | C | Know | Providers assume that all labour ward staff already know how to complete and use a partograph, and perform labour monitoring |  |  |  |
|  | C | Beh Reg | Government accreditation (LaQshya program) mandates routine use of a partograph |  |  |  |
|  | C | Beh Reg | The routine use of a partograph is recommended in best-practice WHO guidelines and policies, which has already been adopted and made mandatory in hospitals |  |  |  |
|  | C | Beh Reg | The WHO guidelines are generic and do no offer recommendations that are specific to the context of individual hospitals |  |  |  |
|  | C | Beh Reg | All hospitals guidelines use consistent definitions of active labour (e.g. starting at 4cm) |  |  |  |
|  | C | Beh Reg | Senior providers ensured junior providers routinely and correctly used the partograph |  |  |  |
|  | O | Env | Limited availability of blank partographs/LCG on the labour ward |  |  |  |
|  | O | Env | Documentation on the LCG was difficult as the boxes were too small |  |  |  |
|  | O | Env | The partograph/LCG is often completed retrospectively |  |  |  |
|  | O | Env | Inadequate staff and high patient numbers made prospective completion of the partograph/LCG difficult |  |  |  |
|  | O | Env | Equipment required to perform clinical measurements was not always available or in working order (e.g. thermometers, fetal dopplers) |  |  |  |
|  | O | Env | Senior providers (e.g. OBGYN) are available to provide support to junior staff in using a partograph |  |  |  |
|  | O | Soc | Senior providers are expected to support the routine use of the partograph and provide supervision to other providers |  |  |  |
|  | M | Bel Cap | Belief that providers were capable of correctly completing a partograph / LCG |  |  |  |
|  | M | Bel Cap | Some providers reported that other staff often left partographs incomplete or incorrectly filled, hence they believed that some staff may lack competence |  |  |  |
|  | M | Bel Con | Belief that the use of a partograph improved women’s health outcomes and experiences of care |  |  |  |
|  | M | Bel Con | Belief that recording numerical measurements on the LCG was more precise and accurate |  |  |  |
|  | M | Bel Con | Perceived LCG completion was more time consuming than previous partograph designs |  |  |  |
|  | M | Bel Con | Concerns that use of the LCG would lead to duplication of work and documentation |  |  |  |
|  | M | Id | Not all cadres of providers are permitted to complete the partograph |  |  |  |
|  | M | Id | Senior providers variably supported the routine use of the partograph |  |  |  |
|  | M | Em | Providers felt the partograph/LCG was easy to use |  |  |  |
|  | M | Em | Providers had a positive attitude towards partograph / LCG use |  |  |  |

| **Appropriate clinical decision making and use of intrapartum *interventions*** | C | Phys | Some providers perceived that a higher skill level is required for LCG interpretation and decision-making |  |  |  |
| --- | --- | --- | --- | --- | --- | --- |
|  | C | Phys | Abnormal findings are recorded on a partograph, but are not always acted on |  |  |  |
|  | C | Know | Belief that a higher professional qualification is required for LCG interpretation and decision-making |  |  |  |
|  | C | Know | Pre-existing provider training on intrapartum care is available at each hospital |  |  |  |
|  | C | Know | Intrapartum training was variable, and not standardised across hospitals |  |  |  |
|  | C | Know | Provider training on intrapartum care is not made available to all cadres |  |  |  |
|  | C | Beh Reg | Providers refer to international and national guidelines or protocols to guide intrapartum decision-making (e.g. WHO, NICE or FOGSI) |  |  |  |
|  | C | Beh Reg | Facility-level guidelines and protocols for intrapartum decision making are not standardised within and across hospitals |  |  |  |
|  | C | Beh Reg | Facility-level guidelines and protocols do not align with current WHO recommendations on LCG use |  |  |  |
|  | C | Beh Reg | Intrapartum practices that WHO recommends against are routine components of care (e.g. routine ARM, routine episiotomy) |  |  |  |
|  | C | Mem | Circling an abnormal result on the LCG is an effective way to promptly identify abnormal findings |  |  |  |
|  | C | Mem | Removal of action and alert lines on LCG was perceived to make clinical decision-making more challenging |  |  |  |
|  | O | Env | All hospitals had 24/7 access to caesarean section |  |  |  |
|  | O | Env | Physical copies of clinical guidelines or policies on intrapartum decision-making were not readily accessible to providers |  |  |  |
|  | O | Env | Hospitals receive a high number of transferred patients, adding complexity in clinical management (i.e. more women arriving late in labour, and more women with intrapartum complications) |  |  |  |
|  | O | Env | Lack of resources dictated intrapartum care options, and what interventions could or could not be performed (e.g. Limited CTG machines reduced capacity to perform inductions) |  |  |  |
|  | O | Soc | Final decision to intervene in labour or birth is made by the most senior provider on duty |  |  |  |
|  | O | Soc | The most senior provider on duty can override any clinical guideline or protocol |  |  |  |
|  | O | Soc | Senior clinicians adhere to the ‘1cm per hour’ rule when making decisions about intrapartum care |  |  |  |
|  | O | Soc | Regular verbal feedback and critique from senior providers guides intrapartum decision-making |  |  |  |
|  | O | Soc | Regular clinical case debriefing with senior providers improved intrapartum decision-making |  |  |  |
|  | M | Bel Cap | Provider belief that there is limited capacity to change intrapartum interventions for patients who have been transferred, where their care has been mostly provided elsewhere |  |  |  |
|  | M | Bel Cap | Providers perceived that removal of action and alert lines would likely make their clinical decision-making more challenging |  |  |  |
|  | M | Bel Cap | Postgraduate doctors did not feel confident in their intrapartum decision making |  |  |  |
|  | M | Bel Con | Provider belief that the use of LCG would reduce unnecessary interventions and therefore reduce workloads |  |  |  |
|  | M | Bel Con | Provider belief that the use of LCG supported and encouraged critical thinking |  |  |  |
|  | M | Bel Con | Belief that LCG and partograph could be used as a communication or handover tool |  |  |  |
|  | M | Id | The most senior clinician on duty is responsible for intrapartum decision making |  |  |  |
|  | M | Id | Confusion about the roles and responsibilities of each cadre in intrapartum decision making and escalation of care |  |  |  |
|  | M | Int | Use of the partograph or LCG as a communication tool |  |  |  |
|  | M | Int | Use of the partograph or LCG as a prompt for referral or patient transfer |  |  |  |

| **Delivery of woman-centred, respectful care** | C | Phys | Formal training to enhance communication skills with patients is not routinely provided for all staff, though some cadres had opportunities. |  |  |  |
| --- | --- | --- | --- | --- | --- | --- |
|  | C | Know | Providers felt they had adequate knowledge about patient communication and consent |  |  |  |
|  | C | Beh Reg | Design of the LCG facilitated communication and clinical handover between staff |  |  |  |
|  | C | Beh Reg | Obtaining consent for procedures is compulsory for providers, and outlined in facility-level guidelines |  |  |  |
|  | O | Env | High workloads made patient communication challenging |  |  |  |
|  | O | Env | Labouring and birthing women shared one large room on labour ward |  |  |  |
|  | O | Env | Privacy during sensitive examinations, such as vaginal examinations, could not be guaranteed |  |  |  |
|  | O | Env | Most hospitals did not have partitions or curtains between beds |  |  |  |
|  | O | Env | Language barriers between providers and patients due to number of local dialects spoken in each region |  |  |  |
|  | M | Bel Cap | Provider belief that delivery of quality intrapartum care is compromised due to high patient to staff ratios |  |  |  |
|  | M | Bel Con | Provider belief that changes to frequency of patient monitoring with LCG use requires additional patient counselling and education |  |  |  |
|  | M | Em | Provider belief that quality of intrapartum care provided to women needs improvement |  |  |  |
|  | M | Em | Most providers believed that communicating with patients during labour and birth was a priority |  |  |  |
|  | M | Em | Most providers are supportive of promoting women-centred care practices |  |  |  |
|  | M | Id | Obtaining consent for procedures is currently the responsibility of doctors only |  |  |  |
|  | M | Id | Patient counselling and education is currently the responsibility of doctors only |  |  |  |
|  | M | Id | Support for nurses and auxillary staff to have a role in patient counselling and education |  |  |  |

| **Labour monitoring education and training** | C | Beh Reg | Providing weekly training and education to labour ward staff is a requirement of hospital accreditation |  |  |  |
| --- | --- | --- | --- | --- | --- | --- |
|  | C | Beh Reg | Individualised and self-directed learning are the most effective modes of partograph training, and could be offered at these hospitals |  |  |  |
|  | O | Env | There are pre-existing education and training sessions on labour monitoring which are part of the specialist OBGYN teaching curriculum |  |  |  |
|  | O | Env | Attendance at labour monitoring training sessions is not compulsory for providers |  |  |  |
|  | O | Env | High workloads prevent providers from attending labour monitoring training sessions |  |  |  |
|  | O | Env | Limited ability to cover staff when attending training sessions prevents staff from attending |  |  |  |
|  | O | Env | All hospitals conduct induction and orientation training for new staff members |  |  |  |
|  | O | Env | Postgraduate doctors on labour room rotate every 3 months – frequent staff turnover presented additional challenges to partograph training and competence |  |  |  |
|  | O | Env | Nurses and nurse-midwives are permanently employed as labour room staff and staff turnover was infrequent |  |  |  |
|  | O | Env | In larger hospitals, training sessions are delivered in ‘batches’ to maximise staff attendance, though required additional effort and resources. |  |  |  |
|  | O | Env | The content, delivery, and timing of partograph training sessions vary across hospitals |  |  |  |
|  | O | Env | Labour monitoring education and training is delivered via didactic lectures |  |  |  |
|  | M | Bel Con | Providers believe that partograph training improves partograph use |  |  |  |
|  | M | Id | Training and education is organised and delivered by senior faculty staff members (OBGYNs) |  |  |  |
|  | M | Em | Provider belief that workshops and seminars are the most effective modes of training |  |  |  |
|  | M | Em | Provider belief that lectures and presentations are the least effective modes of training |  |  |  |
|  | M | Em | Providers want to attend additional training on partograph use and labour monitoring |  |  |  |
|  | M | Em | Provider belief that practical training is vital to support implementation of LCG |  |  |  |
|  | M | Em | Provider belief that future LCG training must cover both completion of LCG and labour monitoring principles more broadly. |  |  |  |

| **Audit and feedback procedures** | C | Beh Reg | Training on Robson Classification of caesarean sections was already included in current curriculum |  |  |  |
| --- | --- | --- | --- | --- | --- | --- |
|  | C | Beh Reg | The use of Robson Classification was not a routine practice within each hospital |  |  |  |
|  | C | Beh Reg | Caesarean section data was not routinely presented as Robson Tables in audit meetings |  |  |  |
|  | O | Env | Regular mortality and morbidity audit meetings were already being conducted at each hospital |  |  |  |
|  | O | Env | The format of these audit meetings was not standardised between hospitals |  |  |  |
|  | O | Env | Caesarean section rates were routinely included in audit meetings |  |  |  |
|  | O | Env | The frequency of audit meetings varied between hospitals |  |  |  |
|  | M | Bel Con | Providers believed that audit and feedback had a positive impact on partograph completion and use |  |  |  |
|  | M | Bel Con | Providers believed that sharing findings of audit and feedback to staff would improve partograph completion and accuracy |  |  |  |
|  | M | Id | Senior faculty providers (OBGYN) were expected to attend all audit meetings |  |  |  |
|  | M | Id | Expected attendance of postgraduate doctors to audit meetings varied between hospitals |  |  |  |
|  | M | Id | Nurse/Nurse-midwives were not expected to attend audit meetings |  |  |  |
|  | M | Id | Audit meetings were led by senior clinicians |  |  |  |

TDF Domains: Know = Knowledge; Phys = Physical skills; Mem = Memory, attention, and decision processes; Beh Reg = Behavioural regulation; Env = Environmental context and resources; Soc = Social Influences; Id = Social/professional role and identity; Bel Cap = Belief about capabilities; Bel Cons = Belief about consequences; Opt = Optimism; Int = Intentions; Goals = Goals; Em = Emotion.
