# Supplementary material for: Developing a Co‐Designed Strategy to Improve Labor Monitoring and Management in India Using the World Health Organization Labour Care Guide: A Mixed‐Methods Formative Study
Source: Birth. 2025 Aug 13;53(1):120–8. doi: 10.1111/birt.70004 (PMC12894475; doi:10.1111/birt.70004)
Supplement: Supplementary file 4 — File S4: birt70004‐sup‐0004‐SupplementaryFile4.docx. [file BIRT-53-120-s002.docx]

**WHO LABOUR CARE GUIDE (LCG) TRAINING AND EDUCATION PROGRAM**

**FACILITATOR’S GUIDE**

*Implementing the WHO Labour Care Guide to reduce the use of Caesarean section in four hospitals in India: a pragmatic, stepped wedge, cluster randomized pilot trial*

**Version**

| **Version** | **Key Changes** | **Date** |
| --- | --- | --- |
| 1.0 | First draft | 02 Aug 2021 |
| 2.0 | Major revisions | 11 Aug 2021 |
| 3.0 | Additional LCG Cases added | 13 Aug 2021 |

1. **BACKGROUND**

An essential component of good-quality intrapartum care is ensuring that women are adequately monitored during labour, such as by prospectively completing a partograph based on regular clinical assessments during labour.

In December 2020, the World Health Organization (WHO) released the new WHO Labour Care Guide (LCG), the “next generation” partograph for all women experiencing labour and birth. The LCG is based on WHO’s latest intrapartum care recommendations which is significantly different from previous partograph designs (such as the WHO simplified partograph). The LCG will be introduced to four high-level obstetric hospitals within Karnataka State, India as part of a stepped wedge cluster randomized pilot trial.

This LCG training program aims to support the implementation of the LCG within routine obstetric practice. LCG training is central to the overall implementation strategy, as its effectiveness in improving clinical outcomes relies on its correct and consistent application within the labour ward setting.

1. **WHO IS THIS MANUAL FOR?**

This manual should be used by the Facility Lead or Facility Co-ordinator who is leading and delivering LCG education and training activities at their hospital. Throughout this LCG Facilitator’s Guide, the Facility Lead and Facility Coordinator will be referred to as the LCG Training Facilitator. Other obstetric faculty staff who are providing or monitoring training activities should also follow this document

1. **HOW SHOULD THIS FACILITATOR’S GUIDE BE USED?**

This facilitator’s guide is a reference for any LCG training facilitator. It ensures **that all minimum required content is covered during LCG training**. Additional materials such as standardized clinical case scenarios, training slide decks, agenda examples and attendance logs are available separately.

As each hospital differs in their workforce, resources and availability, the way in which LCG training is delivered can be adapted to accommodate individual site needs (*see* Section 6). This Facilitator’s Guide provides examples of how LCG training should be provided to maximise LCG implementation at your hospital.

Regardless of how the LCG training program will be adapted at your site, **it is a requirement of all LCG training facilitators to ensure**:

- All components of the LCG (Section 1 – 7) are explained to participants in detail and repeated as necessary.
- Instruction is given to all participants on how to fill out each section of the LCG correctly, using the WHO LCG User’s Manual as a reference guide
- Participants are given multiple opportunities over time to practice filling in an LCG with senior supervision and feedback
- Additional training and support is provided to participants throughout the course of LCG training as needed
- LCG training is interactive, participatory and allows participants to seek support as they need.
- All participants complete pre and post LCG training evaluations.

1. **LCG TRAINING PROGRAM: An Overview**

Once a hospital is randomized, the LCG training program will consist of:

1. **An initial LCG training workshop (2-day workshop)**
2. **Ongoing Case-Based LCG training (8 x weekly sessions)**
3. **Refresher training workshop (0.5 – 1 day as required)**

Facility Leads and Co-ordinators are responsible for ensuring that all LCG training activities are completed and that all necessary staff have attended training. This may mean that **multiple sessions of the LCG workshop may be needed** to allow staff on night or after-hours rosters to attend.

Ongoing case based LCG training should be held during pre-existing allocated time for postgraduate or nursing teaching who are currently working on labour ward. Figure 1 provides a schematic diagram of the LCG training model.

**Figure 1:** LCG Training Program Structure


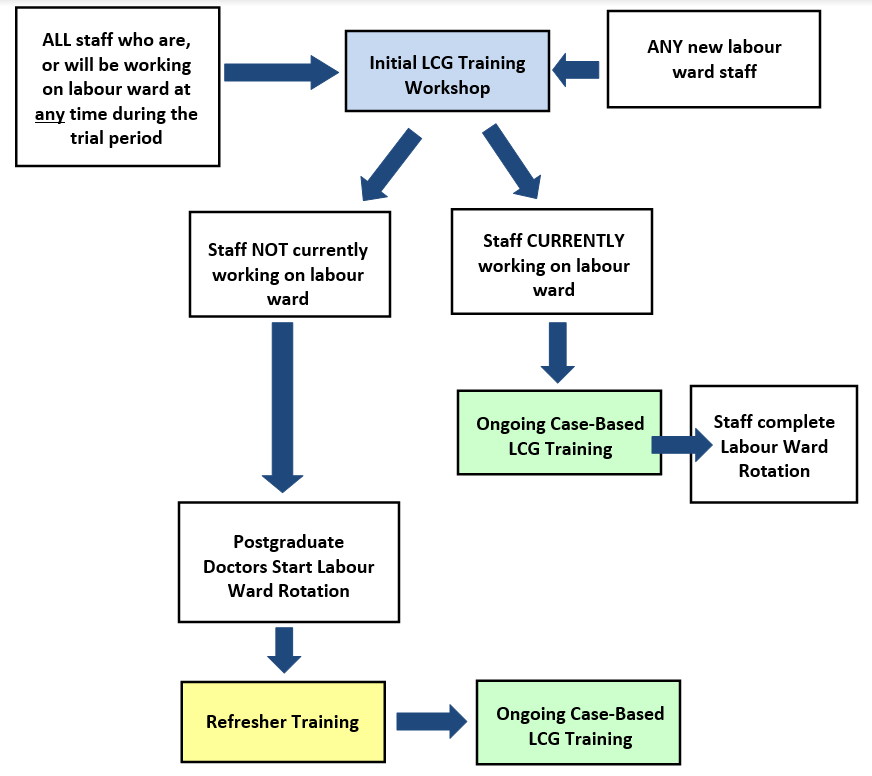


1. **TEACHING-LEARNING METHODS**

Evidence from other types of healthcare provider training show that didactic or lecture-based programs alone have little to no impact on learning outcomes or provider knowledge retention over time. (1-3) Cochrane reviews have shown that more participatory methods such as practical workshops, interactive educational meetings or audit and feedback, can yield improvements in provider knowledge and skills (2,3).

The LCG training program centres around the principles of “*low dose, high frequency*” training – that after participants are taught how to use the LCG (LCG initial workshop), they will then be subject to short but frequent repetitive training (Ongoing Case-Based LCG training) over the course of 8 weeks.

This approach has been shown to increase participant knowledge and retention of knowledge in other areas of obstetric teaching. (4,5).

The following teaching-learning methods are used across the Initial LCG Training Workshop, Ongoing Case-Based LCG Training and Refresher LCG Training activities:

**Lectures**

- Lecture-style format with simplified examples provided throughout.
- Participants are encouraged to ask questions and seek clarification when needed.

**Evolving Clinical Cases**

- A clinical case which is presented in stages by the LCG training facilitator to enable participants to replicate the prospective completion of the LCG.
- As clinical information is revealed, participants should use this information to fill out their own LCG.
- There will be multiple points throughout the clinical cases where all participants can check their LCG documentation is correct. This also provides an opportunity to raise any concerns or queries about LCG documentation.
- At each check point, participants will also be asked to interpret findings and formulate a labour monitoring plan. This will be a group discussion guided by the LCG training facilitator.
- By the end of the clinical case, the participant should have completed the LCG and be provided with an opportunity to reflect on their experiences and any challenges that they faced during completion.

**Small Group Tutorials**

- A small group tutorial is an informal teaching–learning session between a teacher and 2 -5 students.
- Standardized LCG clinical cases have been provided for use in small group settings (*see* Appendix 1), which also give students an opportunity to express their concerns to the teacher and, in turn, give the teacher an opportunity to identify any areas of knowledge which may need more attention.
- Small group tutorials can also be held after bedside teaching sessions as a way to ‘debrief’ and reflect upon their clinical experiences in a supportive setting.

**Bedside teaching**

- LCG training facilitators take small groups of participants into the labour ward to explain and demonstrate how the LCG is completed in ‘real time’.
- Facilitators should directly observe each participant fill out sections of the LCG after participants perform clinical exams or assess maternal-fetal vital status.
- Bedside teaching must be interactive and provide participants with the opportunity to apply their skills, and receive constructive feedback as needed.

**Self-Directed Learning**

- Self-directed learning is when participants are given additional LCG clinical cases to complete in their own time, with answers discussed with the LCG training facilitator.
- This is a useful teaching-learning strategy when additional LCG practice is required; or the facilitator identifies knowledge or skill deficiency within the group.
- Additional cases can be requested from the LCG training team, or facilitators can provide additional training material based on their own clinical cases following approval from the LCG training team.

1. **GETTING STARTED**
   1. **Formulating a Teaching Approach**

It is important for each site to consider the following questions when organising their LCG training program. Each site differs in their resources, number of participants and teaching capacity and so a tailored teaching approach is needed to maximize the number of participants who are able to attend. Please consider the following questions to assist with planning your teaching approach:

*Methods:*

- Modified lectures
- Small group tutorials
- Evolving clinical cases
- Bedside teaching

*Consider:*

- Total number of participants requiring training
- Number of sessions to be held
- Availability of LCG training facilitator(s)
- How many LCG trainers will be available on the day?
- Availability of teaching spaces
- Can bedside teaching be facilitated with number of participants and available LCG training facilitators?

**Initial LCG Training Workshop**

*Methods:*

- Small group tutorials
- Evolving Clinical Cases
- Bedside teaching
- Group and individual feedback

**Ongoing Case-Based LCG Training**

*Consider:*

- Total number of participants at each session?
- How do we ensure relevant staff attend weekly?
- How to integrate into pre-existing teaching time?
- Availability of LCG training facilitator(s)
- Availability of teaching spaces
- Can bedside teaching be facilitated with number of participants and available LCG training facilitators?
- How best to provide feedback and support?
- Will this structure need to change week-to-week?

*Methods:*

- Small group tutorials with postgraduate doctors conducted within pre-existing education time

*Consider:*

- When will these be held for new staff rotating into labour ward?
- When is refresher training feasible for nurses (Gokak only)?
- How do you ensure relevant staff attend?
- How long will training be?
- Availability of LCG training facilitator(s)
- Availability of teaching spaces
- Can bedside teaching be facilitated with number of participants and available LCG training facilitators?
- Feasibility of online participation?

**Refresher LCG Training**

| **Before the workshop:** | Completed? |
| --- | --- |
| - Agree upon the number of workshops required, and the and time/date - Notify all staff of the initial LCG training workshop(s) time/date(s) - Formulate a teaching approach using this Facilitator’s Guide - Formulate an Initial LCG Training Workshop agenda and distribute to all participants - Send a pdf copy of the *LCG User’s Manual* and *LCG Quick Guide* to all participants prior to the workshop (or print so they receive a hard copy prior to the workshop) | - Y - Y - Y - Y - Y |
| **For the Workshop:** |  |
| - Attendance Log to be printed and completed - Blank copies of the LCG (A3 size) to be printed and given to participants - LCG Clinical Cases to be printed and given to participants (if required) | - Y - Y - Y |

- 1. **Initial LCG Training Workshop Checklist**

| **Before Ongoing Case-Based LCG training:** | Completed? |
| --- | --- |
| - Agree to a weekly day/time of each session (ideally within pre-existing teaching) - Identify all eligible participants for the upcoming 8-week block of training (i.e. participants who have attended the Initial LCG training workshop, and/or have attended LCG Refresher training and will be commencing their Labour Ward rotation). - Notify relevant staff of the date/time of weekly sessions, and when their 8-week block will commence. - Formulate a teaching approach using this Facilitator’s Guide - Print hard copies of the *LCG User’s Manual* and *LCG Quick Guide* to keep on the Labour Ward for reference | - Y - Y - Y - Y - Y |
| **For each weekly session:** |  |
| - Attendance Log to be printed - Blank copies of the LCG (A3 size) to be printed and given to participants - LCG Clinical Cases to be printed and given to participants (if required) | - Y - Y - Y |
| **After each weekly session:** |  |
| - Attendance logs to be kept and updated weekly | - Y |

- 1. **Ongoing Case-Based LCG Training Checklist**

| **Before Refresher Training** | Completed? |
| --- | --- |
| - Agree to a day/time when this will occur for all incoming postgraduate doctors or newly employed labour ward staff. - Notify all eligible staff of the date/time of the refresher session - Formulate and distribute an Agenda - Formulate a teaching approach using this Facilitator’s Guide - Ensure all participants receive a copy of the *LCG User’s Manual* and *LCG Quick Guide* before their training. | - Y - Y - Y - Y - Y |
| **For the Refresher Training:** |  |
| - Attendance Log to be printed - Blank copies of the LCG (A3 size) to be printed and given to participants - LCG Clinical Cases to be printed and given to participants (if required) | - Y - Y - Y - Y |

- 1. **Refresher LCG Training Workshop Checklist**

1. **INTIAL LCG TRAINING WORKSHOP**

**Aims:**

1. To promote the use of the WHO LCG as the new labour-monitoring tool in routine clinical practice
2. To ensure all participants are clinically competent in the use, documentation, and interpretation of the WHO LCG

**Objectives:**

On completion of the Initial LCG Training Workshop, participants will be able to:

- Use the WHO LCG as a routine labour-monitoring tool in clinical practice for all births
- Prospectively and accurately document clinical findings on a WHO LCG
- Make sound clinical decisions based on the interpretation of the WHO LCG
- Implement best-practice supportive care principles into routine clinical practice

**Plan:**

- A two- day clinical workshop which covers each section of the WHO LCG in detail and provides opportunity for participants to practice LCG completion with supportive supervision and feedback.
- The workshop may be repeated to ensure all participants have had LCG training.

**Resources:**

- World Health Organization (WHO) Labour Care Guide User’s Manual.
- World Health Organization (WHO) Intrapartum Care Recommendations 2018
- LCG Quick Guide Clinical Cases
- Blank LCGs (A3 size)
- WHO LCG slide deck.

The introduction of the WHO LCG requires clinicians to use new definitions of active labour onset and to become comfortable with allowing women to remain in first stage of active labour for longer periods of time (permitting no evidence of maternal-foetal compromise).

Key differences in clinical practice which should be emphasised including:

- The LCG should be commenced once a woman is in the first stage of active labour. **Active labour is now considered to be from 5cm of cervical dilation instead of 4cm**.
- **Supportive care practices** including the inclusion of labour companion, allowing women to eat and drink, be mobile during labour and have access to analgesia should be routine additions to labour management. These practices should be supported on a hospital-policy level and recorded within the LCG.
- The LCG should be completed **prospectively**
- We **no longer use the 1cm/hour rule** for cervical dilation as we know this is unrealistically fast for most women and may increase the use of unnecessary labour intervention.
- Time limits for each stage of cervical dilation are noted on the LCG. In the absence of maternal-fetal compromise, these limits can be used as a guide to assess labour progress.
- **The LCG does NOT replace clinical judgement**. It is a labour-monitoring tool which should aid clinical decision making.
- The LCG is intended for use in low-risk women, including low-risk multiples planning on a vaginal delivery. High-risk women may need additional monitoring.
- Women should **still be monitored during the latent phase**, with assessment documented within the patient’s medical records.
- **More time is given to women in the second stage of labour as long as there are no signs of maternal or fetal compromise** – for primip, allow three hours; for multips, allow two hours.

**
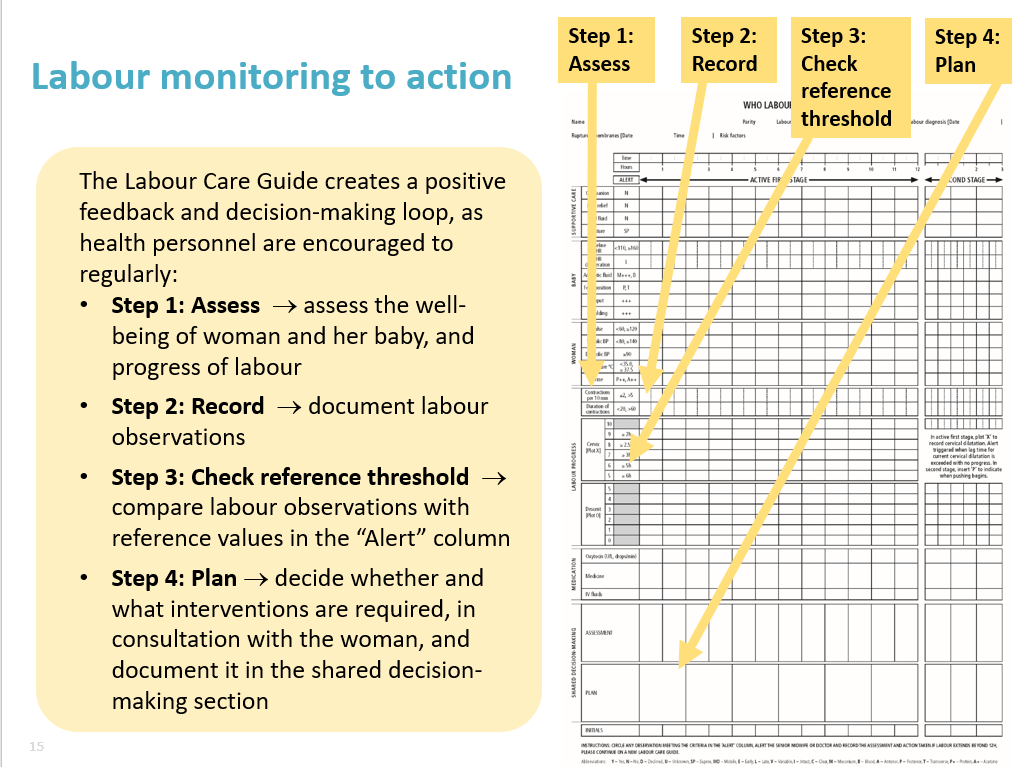
**A slide deck will be provided to all LCG training facilitators, which contains an explanation of each LCG component in detail with examples. The LCG slide decks can be adapted according to your hospital site’s teaching plan and workshop structure. Once each section of the LCG is explained in detail, LCG training facilitators should emphasise how the clinical assessments recorded within the LCG should be interpreted and prompt safe clinical action (or intervention if needed). In the LCG user’s Manual, you will see reference to the ***Assess 🡪 Record 🡪 Check 🡪 Plan*** framework.

**Common Q&As to consider:**

***For whom should the LCG be used?*** All women in labour who are planning a vaginal delivery. High-risk women may require additional monitoring and care.

***When should the LCG be initiated?*** When women have entered the active phase of the first stage of labour (i.e., cervical dilatation of 5 cm or more).

***Where should the LCG be used?*** The LCG is designed for use at all levels of care in health facilities.

***Can the LCG be used for twins if vaginal birth is attempted?*** Yes, the LCG can be used for low-risk multiple deliveries (e.g., Cephalic DCDA twins, where previous NVD has been achieved). Use one LCG per infant for monitoring and clearly document ‘Twin 1’ and ‘Twin 2’

***There is not enough space in the boxes of the LCG, what do I do?*** The LCG are printed on an A3 page to make the boxes as large as possible. Any additional information can be documented within the patient’s medical notes. The LCG is meant to be a supplement and used in conjunction with the medical records – it should not be a replacement.

***It is difficult to document time when more frequent/additional assessment is required, what should I do?*** Avoid pre-filling in the time scale on the top of the LCG. This allows for clear documentation of exact time if any additional assessments are required, and this can be prospectively filled in. Alternatively, a clinician can document in Section 7 (Plan & Assessment) that an additional assessment has been performed and then document the findings within the patient’s medical records.

***How do I monitor women in the latent phase of active labour (i.e., before 5cm of cervical dilation)?*** While the LCG does not need to be commenced before this time, women presenting in latent phase of labour still need to be regularly monitored and assessed for signs of maternal-foetal compromise. Women who are being offered an induction of labour should be assessed in accordance with local clinical guidelines. Women presenting in spontaneous labour should be assessed as clinical need dictates.

***Allowing women more time to dilate in the active phase of labour seems risky. Would this not increase her risk of obstructed labour?*** The LCG was developed because of new knowledge that current labour curves do not truly reflect the variability associated with labour progression in women who are low-risk and go on to have an uncomplicated delivery. Labour progress should always be assessed prospectively, so that the clinician also incorporates current maternal-fetal vital status in clinical decision making. If progress is slow but normal (as indicated by the LCG) and there are no signs of maternal-fetal compromise, then it would be appropriate to continue monitoring. If progress is slow but there are signs of maternal-fetal compromise, then intervention is required. **The LCG does not replace clinical judgement.**

***Two to three hours seems too long for second stage. Would this be too risky?***

**The LCG does not replace a clinician’s judgement** and progression of second stage should be assessed with maternal-fetal vital status in mind. This recommendation applies to all low-risk women, where there are no signs of maternal-fetal compromise following frequent assessment. If there are any doubts as to whether clinical intervention is required, consultation with a senior clinician is recommended.

- 1. **Example Workshop Agenda**

| **DAY 1** | **Lecturer/Workstations** | **Recommended Time** |
| --- | --- | --- |
| 09: 00 – 09: 20 | Registration of attendance | 20 minutes |
| 09:30 – 10: 00 | Welcome  Pre- LCG Training Evaluation completion  Key Documents & Reference Materials  LCG Training Program Overview | 30 minutes |
| 10:00 – 11:15 | - Introduction to the WHO Labour Care Guide - Supportive Care Principles - WHO LCG v Simplified Partograph | 75 minutes |
| 11:15 -11:30 | *Tea Break* | 15 minutes |
| 11:30 – 12: 15 | Completing the Labour Care Guide:   - Section 1 & 2 - Discussion and Case Example(s) | 45 minutes |
| 12:15 – 13:00 | Completing the Labour Care Guide:   - Section 3 & 4 - Discussion and Case Example(s) | 45 minutes |
| 13:00 – 13:50 | *Lunch* | 50 minutes |
| 13:50 – 14:45 | Completing the Labour Care Guide:   - Section 5 - Discussion and Case Example(s) | 45 - 60 minutes |
| 14:45 – 15:30 | Completing the Labour Care Guide:   - Section 6 & 7 - Discussion and Case Example(s) | 45 minutes |
| 15:30 – 15:45 | *Tea Break* | 15 minutes |
| 15:45 – 17:00 | - Evolving Clinical Case (“Mrs Annurada at 38 weeks gestation”) - Discussion   *Meeting close* | 75 minutes |

| **DAY 2** | **Lecturer/Workstations** | **Recommended Time** |
| --- | --- | --- |
| 09:00 – 09:20 | Registration of attendance | 20 minutes |
| 09:20 – 09: 40 | Welcome  Explanation of Day 2 activities  Participants divided into small tutorial groups | 20 minutes |
| 09:40 – 10:45 | Self-directed learning and discussion:   - Participants given LCG Clinical Case to complete by-themselves - Discussion and Feedback in small tutorial groups | 60 minutes |
| 10:45 – 11:00 | *Tea Break* | 15 minutes |
| 11: 00 –  12: 15 | Small Group Work Tutorial: LCG Clinical Cases 1 & 2   - LCG completion - Discussion and feedback | 75 minutes |
| 12:15 | *Lunch* | 45 minutes |
| 13:00 – 14:15 | Small Group Bedside Teaching   - LCG completion prospectively on labour ward - Discussion & reflection in small groups | 75 minutes |
| 14:15 – 14:30 | *Tea Break* | 15 minutes |
| 14:30 – 15:15 | Small Group Tutorial: LCG Clinical Case 3   - LCG completion - Discussion and feedback | 45 minutes |
| 15:15 – 16:00 | Post- LCG Training Evaluation Completion & *Meeting Close* | 40 minutes |

- 1. **Attendance Log**

| **Initial LCG Training Workshop** | | | **Date:___________** | **Date:________** |
| --- | --- | --- | --- | --- |
| **Participant ID**  (Medical Council Registration Number / Nursing Council Registration Number) | **Department** | **Position** | **Attendance**  **(Day 1)** | **Attendance**  **(Day 2)** |
|  |  |  |  |  |
|  |  |  |  |  |
|  |  |  |  |  |
|  |  |  |  |  |
|  |  |  |  |  |
|  |  |  |  |  |
|  |  |  |  |  |
|  |  |  |  |  |
|  |  |  |  |  |
|  |  |  |  |  |
|  |  |  |  |  |
|  |  |  |  |  |
|  |  |  |  |  |
|  |  |  |  |  |
|  |  |  |  |  |
|  |  |  |  |  |
|  |  |  |  |  |
|  |  |  |  |  |
|  |  |  |  |  |
|  |  |  |  |  |
|  |  |  |  |  |
|  |  |  |  |  |
|  |  |  |  |  |
|  |  |  |  |  |

- 1. **Initial LCG Training Workshop: Evolving Clinical Case**

**Aims:**

1. To demonstrate how the LCG should be prospectively used and what it looks like when completed
2. To practice LCG completion
3. To discuss challenges faced with LCG completion and reflect on LCG completion skills

**Objectives:**

Following completion of this evolving clinical case, participants will be able to:

- Correctly document clinical findings onto the LCG using the recommended abbreviations and key
- Recognize abnormal clinical values and how to document clinical decision making
- Understand how the new time scales are used for monitoring labour progress
- Refer to supplementary resources such as the WHO LCG User’s Manual to assist with LCG completion

**Plan:**

An evolving clinical case which is presented by the LCG training facilitator(s). This can be presented to the entire group of workshop participants, or participants can be divided into smaller groups. The presentation will be provided on power point slide to LCG training facilitators.

**Resources:**

- World Health Organization (WHO) Labour Care Guide User’s Manual.
- Evolving Clinical Case Slide deck – “*Mrs Annurada Gowravi presents in labour at 38 weeks gestation*”
- Blank copies of the LCG (A3 size) for participants.

The Evolving Clinical Case slide deck *(“Mrs Annurada Gowravi presents in labour at 38 weeks gestation”*) will be provided in full to all LCG training facilitators for use. Participants will be provided with their own blank copies of the LCG (A3 size) which they will fill out as the clinical information is revealed. There will be clinical checkpoints where the case will be paused, and participants will have the opportunity to compare their completed LCGs with the LCG training Facilitator’s answer.

**LCG Training Facilitator tips:**

- Ensure ample time is given to participants to transcribe clinical information
- Encourage discussion at every clinical “Check Point”
- Take note of any areas that have been challenging to participants – this may require further training or review in subsequent training sessions. Please discuss this with the central LCG training team so that we can provide you with support in additional training activities if needed.
  1. **Initial LCG Training Workshop: Small Group Tutorials**

**Aim:**

1. To practice additional LCG completion with clinical scenarios
2. To discuss each case with the support of a small group of peers

**Objectives:**

Following the completion of small group tutorials, participants will be able to:

- Complete the LCG and apply their knowledge to a diverse range of clinical scenarios
- Seek feedback and discuss any concerns they have with LCG completion
- Assist peers with LCG completion

**Plan:**

Divide the participants into small groups (depending on the number of participants and available LCG training facilitators). Have the participants work through the LCG clinical cases, and discuss completion once done in a group setting.

**Resources:**

- World Health Organization (WHO) Labour Care Guide User’s Manual.
- LCG Clinical Cases (*See* Appendix 1)
- Additional Cases as created by the LCG training facilitator (optional)
- Blank copies of the LCG (A3 size) for participants.

For a large class, consider dividing participants into smaller groups to discuss LCG clinical cases. Example LCG Clinical Cases have been provided for your use in these practical sessions, but LCG training facilitators may consider creating their own examples or using retrospective de-identified cases notes to reflect typical patient characteristics at their local sites.

**LCG training facilitator tips for small group work tutorials:**

- Consider the ratio of LCG training facilitator(s) to number of participants prior to the workshop to ensure this model of teaching would be appropriate.
- Decide before the workshop whether participants are to complete the LCG themselves first, and then go through the case as a group OR the LCG is to be completed together as a small group.
- Allow ample time for discussions and questions
- Where possible, allow other group participants to try and answer questions or help solve any challenges – this promotes team building and also solidifies learning for all.
  1. **Initial LCG Training Workshop: Bedside Teaching**

**Aim:**

1. To demonstrate how the LCG is to be completed in ‘real-life’
2. To identify and address challenges faced when completing the LCG by the patient bedside
3. To demonstrate how the LCG is interpreted and can guide clinical decision making

**Objectives:**

Following the completion of bedside teaching, participants will be able to:

- Complete the LCG prospectively in the labour ward environment
- Use the LCG to guide clinical decision making, to avoid unnecessary labour intervention

**Plan:**

In small groups, LCG training facilitators and/or senior clinicians will demonstrate the use of the LCG in real-time. Verbal permission will be sought from the labouring woman prior to bedside teaching.

**Resources:**

- World Health Organization (WHO) Labour Care Guide User’s Manual.
- Blank copies of the LCG (A3 size) for participants.

LCG training facilitators are encouraged to provided bedside LCG teaching. This may depend on the number of participants, availability of facilitators and the appropriate acuity of patients on the labour ward. Ensure verbal consent from the labouring woman is obtained prior to conducting the bedside teaching session.

Bedside teaching is a good opportunity to demonstrate not only how the LCG is completed prospectively (in real time), but how to identify and address any queries that arise when confronted with “real life” examples that do not perfectly fit in a pre-scripted scenario. Bedside teaching can be integrated within the Initial LCG training workshop and used as an adjunct to ongoing LCG clinical training.

**Tips of LCG training facilitators:**

- If possible, find a low-risk clinical case with normal progression of labour. When learning a new skill, learning on ‘the normal case’ is an important foundation.
- If women are postpartum, retrospective clinical notes could be used as a discussion point and LCGs completed as part of a small group exercise.
- Allow ample time for discussion and questions.

1. **ONGOING LCG CASE-BASED LEARNING**

**Aim:**

1. To reinforce LCG knowledge and skills over time.
2. To provide weekly practice at completing an LCG with supportive supervision.

**Objectives:**

Following completion of the ongoing LCG case-based learning sessions, participants will be able to:

- Use the LCG correctly as part of their routine labour monitoring
- Retain LCG knowledge and skills throughout their labour ward rotation
- Maintain a high level of accuracy in LCG documentation and completion
- Correctly interpret findings of the LCG to inform clinical decision making

**Plan:**

LCG training facilitators are to conduct eight weekly LCG training sessions, which will be integrated within pre-existing education time. Each session will be 20 minutes in length.

**Resources:**

- World Health Organization (WHO) *Labour Care Guide User’s Manual.*
- LCG Clinical Cases (*See* Appendix 1)
- Additional Cases as created by the LCG training facilitator (optional, and subject to discussion with the LCG training team)
- Blank copies of the LCG (A3 size) for participants.

Once all participants have completed the Initial LCG training, the ongoing LCG case-based training can start and should include:

- Postgraduate doctors who are currently working on labour ward as part of their 3-month rotation
- Postgraduate doctors who may have occasional shifts on labour ward during the 3-month term (e.g., staff rostered to OPD, but will be called to work on labour ward if busy)
- Nurses who permanently work on the labour ward, and any nurses that may have occasional shifts on labour ward when required (Gokak only).

**LCG case-based learning can start as soon as all labour ward staff have completed their Initial LCG Training Workshop.**

**Tips of LCG training facilitators:**

- One LCG clinical case should be discussed each week, for eight weeks.
- LCG clinical cases have been designed so that they can be presented to participants as an evolving clinical case where the LCG is completed as a group; OR the clinical cases can be completed by participants individually at the start of the session and then completed LCGs can be discussed amongst the group.
- Allow ample time for discussions and questions
- Where possible, allow other group participants to try and answer questions or help solve any challenges – this promotes team building and solidifies learning for all.
- Consider the ratio of LCG training facilitator(s) to number of participants prior to the workshop to ensure this model of teaching would be appropriate.

LCG training facilitators can provide participants with additional LCG training on the labour ward in addition to these sessions. For example, LCG training facilitators may directly supervise LCG completion on the labour ward and provide informal feedback to students.

However, ongoing LCG clinical cases sessions also provide an opportunity for LCG training facilitators to identify areas of LCG knowledge or skill deficiency which need to be rectified.

1. **LCG REFRESHER TRAINING WORKSHOP**

**Aim:**

1. To provide refresher training to postgraduate doctors who completed their Initial LCG training workshop > 3 months ago.

**Objectives:**

Following completion of the LCG Refresher training, participants will be able to:

- Use the LCG correctly as part of their routine labour monitoring
- Start ongoing LCG case-based training as part of their labour ward rotation
- Maintain a high level of accuracy in LCG documentation and completion
- Correctly interpret findings of the LCG to inform clinical decision making

**Plan:**

The LCG refresher training is a “mini” version of the Initial LCG training workshop. Participants have attended the initial LCG training workshop but have not used the LCG in routine clinical practice yet (i.e., have not started their labour ward rotation).

**Resources:**

- World Health Organization (WHO) Labour Care Guide User’s Manual.
- LCG Clinical Cases (*See* Appendix 1)
- Additional Cases as created by the LCG training facilitator (optional)
- Blank copies of the LCG (A3 size) for participants.

The LCG refresher training workshop is to be held in 3-month blocks to coincide with the new rotation of postgraduate doctors onto the labour ward. As these participants should have completed the Initial LCG training workshop, half a day will be all that is required and will depend on the preference and availability of LCG training facilitators. An attendance record must be kept, and like the Initial LCG training workshop, an agenda and combination of learning activities can be developed to maximise learning.

**9.1 Example of Refresher LCG Training Agenda**

| **DAY 1** | **Lecturer/Workstations** | **Recommended Time** |
| --- | --- | --- |
| 09: 00 – 09: 20 | Registration of attendance | 20 minutes |
| 09:30 – 10: 00 | Welcome  Pre- LCG Training Evaluation completion  Key Documents & Reference Materials  LCG Training Program Overview | 30 minutes |
| 10:00 – 11:15 | - Introduction to the WHO Labour Care Guide - Supportive Care Principles - WHO LCG v Simplified Partograph | 45 minutes |
| 11:15 -1200 | Completing the Labour Care Guide: Section 1 & 2   - Discussion and Case Example(s) | 45 minutes |
| 12:00 – 12: 15 | *Tea Break* | 45 minutes |
| 12:15 – 13:00 | Completing the Labour Care Guide:   - Section 3 & 4 - Discussion and Case Example(s) | 45 minutes |
| 13:00 – 13:50 | *Lunch* | 50 minutes |
| 13:50 – 14:45 | Completing the Labour Care Guide:   - Section 5 - Discussion and Case Example(s) | 45 - 60 minutes |
| 14:45 – 15:30 | Completing the Labour Care Guide:   - Section 6 & 7 - Discussion and Case Example(s) | 45 minutes |
| 15:30 – 15:45 | *Tea Break* | 15 minutes |
| 15:45 – 17:00 | - Evolving Clinical Case (“Mrs Annurada at 38 weeks gestation”) - Discussion   *Meeting close* | 75 minutes |

**REFERENCES**

1. Bluestone J, Johnson P, Fullerton J, Carr C, Alderman J, BonTempo J. Effective in-service training design and delivery: evidence from an integrative literature review. Hum Resour Health. 2013; 11:51.
2. Ivers N, Jamtvedt G, Flottorp S, Young JM, Odgaard-Jensen J, French SD, et al. Audit and feedback: effects on professional practice and healthcare outcomes. Cochrane Database Syst Rev. 2012(6):CD000259.
3. Forsetlund L, Bjorndal A, Rashidian A, Jamtvedt G, Brien MA, Wolf F, et al. Continuing education meetings and workshops: effects on professional practice and health care outcomes. Cochrane Database Syst Rev. 2009(2):CD003030.
4. Jhepigo. Low Dose, High Frequency: A Learning approach to improve health workforce competence, confidence, and performance [Internet]. 2013 Aug 11 [cited 2021 Apr 20]. Available from: http://hms.jhepigo.org/wp-content/uploads/2016/08/LDHF_briefer.pdf
5. Atukunda IT, Conecker GA. Effect of a low-dose, high-frequency training approach on stillbirths and early neonatal deaths: a before-and-after study in 12 districts of Uganda. Lancet Glob Heal. 2017;5:S12.

**LCG CLINICAL CASE #1**

**Case 1.** *Ms Rumi Jones is a 27-year-old G3P1 at 37 ^+ 2^ weeks gestation on 4^th^ March 2021 at 0600.*

She has had regular antenatal care and an uncomplicated pregnancy. She is taking oral iron supplementations for mild iron deficiency anaemia. Rumi has nil significant past medical or surgical history. She is Rhesus positive, and the placenta was clear from the os at anatomy ultrasound.

Rumi reports that she has had clear vaginal loss for the last 2 hours and is now experiencing regular abdominal and back pain, which is getting more intense. She feels the baby moving.

The midwife in charge of the admission offers Rumi a clinical evaluation to assess fetal well-being and labour stage.

*Please complete each section of the LCG based on the clinical findings. Please use the LCG User’s Manual as a guide as required.*

|  | **Fetal Assessment** | | | | | |
| --- | --- | --- | --- | --- | --- | --- |
|  | **Baseline FHR** | **FHR Deceleration** | **Amniotic Fluid** | **Fetal**  **Position** | **Caput** | **Moulding** |
| 0600 | 145 | No | Membranes Ruptured – clear liqor | Occiput posterior | None | Sutures apposed |
| 0630 | 138 | No | **-** | - | - | - |
| 0700 | 123 | No | **-** | - | - | - |
| 0730 | 154 | No | - | - | - | - |
| 0800 | 155 | No | **-** | - | - | - |
| 0830 | 132 | Early | - | - | - | - |
| 0900 | 117 | Early | - | - | - | - |
| 0930 | 126 | No | - | - | - | - |
| 1000 | 135 | Early | Blood stained liqor | Occiput posterior | Mild | Sutures apposed |

|  | **Maternal Vital Assessment** | | | | |
| --- | --- | --- | --- | --- | --- |
|  | Pulse | Systolic BP | Diastolic BP | Temperature ^o^C | Urine |
| 0600 | 80 | 124 | 80 | 37.3 | Clear |
| 0700 | 80 | 117 | 81 | 37.2 | - |
| 0800 | 78 | 125 | 74 | 37.1 | - |
| 0900 | 81 | 127 | 80 | 37.2 | - |
| 1000 | 83 | 130 | 84 | 36.9 | Clear |

|  | **Labour Progress** | | | |
| --- | --- | --- | --- | --- |
|  | **Cervix** | **Descent** | **Contractions**  **per 10 minutes** | **Duration of contractions** |
| 0600 | 6cm | 4/5 | 3 | 30 |
| 0630 | - | - | 3 | 30 |
| 0700 | - | - | 3 | 40 |
| 0730 | - | - | 3 | 30 |
| 0800 | - | - | 4 | 45 |
| 0830 | - | - | 4 | 40 |
| 0900 | - | - | 4 | 45 |
| 0930 | - | - | 4 | 60 |
| 1000 | 9cm | 2/5 | 4 | 60 |

|  | **Supportive Care Practices** | | | |
| --- | --- | --- | --- | --- |
|  | **Companion** | **Pain Relief** | **Oral Fluid** | **Posture** |
| 1100 | Yes | IV Opioid | Declined | Supine |
| 1200 | Yes | Declined | Declined | Supine |

**Q:** After your assessment of Ms Jones at 10am, what would your next action and ongoing monitoring plan be? Discuss your answer and provide support for your clinical action.

**LCG Training Facilitators:**

- The clinical concern is the presence of blood-stained liqor on examination.
- There is otherwise no suggestion of maternal or fetal compromise.
- Prompt students to consider their options – they include delivery, more frequent monitoring (CTG may be appropriate, or increased auscultation), shorten time to next review (e.g., in 1 hour rather than 2), discuss with senior or continue.
- Prompt students to consider any ‘red flag’ symptoms which may change how the LCG findings are interpreted (e.g. abdominal pain during contractions, more PV bleeding etc).

After discussion with the OBGYN on labour ward, a decision was made to continue to monitor Ms Jones and reassess her in 1 hour. She has been told of the plan and has agreed and the nurses have been instructed to call if there have been any changes to her clinical condition in the meantime. *Please complete the LCG with the following observations.*

|  | **Fetal Assessment** | | | | | |
| --- | --- | --- | --- | --- | --- | --- |
|  | **Baseline FHR** | **FHR Deceleration** | **Amniotic Fluid** | **Fetal**  **Position** | **Caput** | **Moulding** |
| 1030 | 151 | No |  |  |  |  |
| 1100 | 138 | No | **-** | - | - | - |
| 1130 | 146 | No | **-** | - | - | - |
| 1200 | 155 | No | Blood stained | LOT | ++ | + |

|  | **Maternal Vital Assessment** | | | | |
| --- | --- | --- | --- | --- | --- |
|  | Pulse | Systolic BP | Diastolic BP | Temperature ^o^C | Urine |
| 1030 |  |  | - | - | - |
| 1100 | 90 | 130 | 90 | 37.4 | Not done |
| 1130 |  |  | - | - | - |
| 1200 | 100 | 128 | 87 | 37.4 | Not done |

Mrs Jones is fully dilated and feels the urge to push. She is encouraged to do so with each contraction.

**Q.** How will you continue to monitor Mrs Jones throughout the second stage?

*Please fill out the LCG using the following parameters for second stage*

|  | **Labour Progress** | | | |
| --- | --- | --- | --- | --- |
|  | **Cervix** | **Descent** | **Contractions**  **per 10 minutes** | **Duration of contractions** |
| 1030 |  |  | 3 | 30 |
| 1100 | - | - | 3 | 30 |
| 1130 | - | - | 3 | 40 |
| 1200 | 10cm | 0/5 | 4 | 40 |

| **Time** | **FHR** | **FHR Deceleration** | **Contractions** | **Duration** |
| --- | --- | --- | --- | --- |
| 12:00 | 155 | No | 4 | 40 |
| 12:05 | 157 | No | 4 | 40 |
| 12:10 | 163 | No | 4 | 40 |
| 12:15 | 166 | No | 4 | 40 |
| 12:20 | 167 | Late | 4 | 40 |
| 12:25 | 169 | Late | 4 | 50 |
| 12:27 | 155 | Late | 4 | 50 |

The postgraduate doctor performed an episiotomy and vacuum extraction. A live male infant was born at 12:27 with Apgar scores of 7 and 9 at 5 minutes.

**LCG CLINICAL CASE #2**

**Case 2:** *Ms Shrimati is a 20-year-old G2P1 at 37 weeks gestation.*

She presents with worsening abdominal pain and has been “leaking fluid” per vaginum since 1000 this morning. It is now 22:45. Ms Shrimati was asked about her previous obstetric history, and her current pregnancy:

- Previous vacuum delivery of a 3.2kg infant in 2018
- Complicated by a PPH of 700mL requiring administration of uterotonics.
- Regular antenatal clinic attendance. No complications this pregnancy
- No significant past of family history.

The postgraduate doctor explained that an examination, including vaginal examination, would be required to determine how far her labour has progressed. Ms Shrimati agrees to proceed. She is offered pain relief and the option to bring a labour companion to accompany her, but she declines both.

*Please complete your LCG using the clinical information provided.*

**On Admission: 24^th^ March, 2021 at 2245**

Maternal examination findings:

- No pallor or oedema
- Pulse = 80bpm
- BP = 120/75
- Temp = 36.4^o^C / 97.5 F
- Urine = No protein or acetone

Obstetric examination findings:

- Longitudinal lie, cephalic
- Contractions – 3:10, lasting 40 seconds.
- Cervix - 6cm dilated.
- Fetal presentation – ROA
- Fetal Descent – at spines (0) / Engagement = 3/5^th^ palpable
- Membranes absent – clear liqor
- Nil caput or moulding

Fetal Examination findings:

- FHR = 125 bpm, no decelerations

**Shared Decision Making**

Assessment

- Ms Shrimati is in active labour
- No signs of maternal or fetal distress
- But ROM > 12 hours

Plan

- Initiate the LCG.
- Re-examine in 4 hours, unless change in clinical condition.
- Fetal movements have been normal today.

**The next 4 hours**

*March 24^th^, 2021 at 22:45 – March 25^th^ 2021, at 01:45*

Section 2: Supportive Care

|  | 22:45 | 23:45 | 00:45 | 01:45 |
| --- | --- | --- | --- | --- |
| Companion | Declined | Declined | Declined | Yes |
| Pain Relief | Declined | Not Offered | Yes | Declined |
| Oral Fluid | Water | Not Offered | Declined | Water |
| Posture | Pacing | Supine | Mobile | Mobile |

Section 3: Baby

|  | 22:45 | 23:15 | 23:45 | 00:15 | 00:45 | 01:15 | 01:45 | 02:15 |
| --- | --- | --- | --- | --- | --- | --- | --- | --- |
| FHR | 125 | 146 | 138 | 149 | 150 | 125 | 134 | 147 |
| FHR Deceleration | N | N | N | V | N | N | V | V |

Section 5: Labour Progress (Contractions)

|  | 22:45 | 23:15 | 23:45 | 00:15 | 00:45 | 01:15 | 01:45 | 02:15 |
| --- | --- | --- | --- | --- | --- | --- | --- | --- |
| Contractions per 10 mins | 3 | 3 | 3 | 3 | 4 | 4 | 3 | 2 |
| Duration | 40 | 40 | 30 | 30 | 30 | 40 | 30 | 30 |

**Shared Decision Making**

Assessment

- No signs of maternal or fetal distress
- Contraction frequency, duration and strength are reducing.

Plan

- Re-examine now as planned (02:45).
- Consider augmentation with Oxytocin.

**Re-examination at 4 hours.**

25^th^ March 2021 at 02:45

Ms Shrimati has received IV Tramadol at 0045.

At 0245, she is walking around the room and her husband, Abir, has arrived to support her. She has declined oral intake.

Maternal examination findings:

- No pallor or oedema
- Pulse = 95bpm
- BP = 128/75
- Temp = 36.4^o^C / 97.5 F
- Urine = No protein or acetone

Obstetric examination findings:

- Longitudinal lie, cephalic
- Contractions – 3:10, lasting 30 seconds.
- Cervix – 7cm
- Fetal presentation – ROA
- Engagement – 2/5^th^ palpable
- Liqor is still clear.
- Caput + / Molding +

Fetal Examination findings:

**Shared Decision Making**

Assessment

- No signs of maternal or fetal distress
- Contraction frequency, duration and strength are reducing.
- Labour is still progressing within recommended timeframe.

Plan

- Re-examine in 2 hours (04:45)
- Commence Oxytocin for augmentation.
- CTG on for monitoring
- FHR = 137 bpm, no decelerations

**The next 2 hours**

*25^th^ March 2021 from 0245 – 04:45*

Section 2: Supportive Care

|  | 02:45 | 03:45 | 04:45 |
| --- | --- | --- | --- |
| Companion | Yes | Yes | Yes |
| Pain Relief | Declined | Not Offered | Declined |
| Oral Fluid | Declined | Not Offered | Declined |
| Posture | Pacing | Supine | Supine |

Section 3: Baby

|  | 02:45 | 03:15 | 03:45 | 04:15 | 04:45 |
| --- | --- | --- | --- | --- | --- |
| FHR | 137 | 148 | 130 | 150 | 153 |
| FHR Deceleration | N | N | V | N | N |

Section 5: Labour Progress (Contractions)

|  | 02:45 | 03:15 | 03:45 | 04:15 | 04:45 |
| --- | --- | --- | --- | --- | --- |
| Contractions per 10 mins | 3 | 3 | 3 | 4 | 4 |
| Duration | 30 | 40 | 40 | 40 | 40 |

Section 6: Medications (Oxytocin)

|  | 02:45 | 03:45 | 04:45 |
| --- | --- | --- | --- |
| Oxytocin (mU/mL) | 6 | 12 | 24 |

**Next Planned Examination**

*March 25^th^ 2021 at 04:45*

Ms Shrimati is sitting in the bed with CTG monitoring on as per hospital protocol. There are no signs of fetal or maternal distress, and she is now having strong 4:10 contractions lasting 40 seconds. She tells the postgraduate doctor that she feels pressure and an urge to push.

Maternal examination findings:

- No pallor or oedema
- Pulse = 100 bpm
- BP = 130/75
- Temp = 36.4^o^C / 97.5 F
- Urine = No protein or acetone

Obstetric examination findings:

- Fetal descent = -2 station
- Cervix – 10cm
- Fetal presentation – OA
- Liqor is still clear.
- Caput ++ / Molding +

Fetal Examination findings:

- FHR = 153 bpm, no decelerations

**Shared Decision Making**

Assessment

- Second Stage Initiated
- No signs of maternal or fetal distress

Plan

- Continue CTG
- To support pushing as she feels the urge and is fully dilated.

**Second Stage**

*25^th^ March, 2021 at 0500*

Section 3: Baby

|  | 05:00 | 05:15 | 05:30 | 05:45 | 05:56 |
| --- | --- | --- | --- | --- | --- |
| FHR | 159 | 138 | 163 | 153 | Birth |
| FHR Deceleration | N | V | V | V | Birth |

Section 5: Labour Progress (Contractions)

|  | 05:00 | 05:15 | 05:30 | 05:45 | 05:56 |
| --- | --- | --- | --- | --- | --- |
| Contractions per 10 mins | 4 | 4 | 4 | 4 | Birth |
| Duration | 40 | 40 | 40 | 40 | Birth |

**LCG CLINICAL CASE # 3**

***Case 3.*** Mrs Prisha Anand is a 32-year-old G7P6^-1^ presenting to the labour ward on 28^th^ August 2020 with increasing abdominal pain. She has had limited antenatal care this pregnancy. Her gestation is approximately 41 weeks based on last menstrual period. She has had six precipitous vaginal births, with her last infant stillborn at 35 weeks gestation in 2018. Prisha had been experiencing abdominal pain for the past 48 hours but the pain has now become unbearable to manage at home. Prisha reports reduced fetal movements since last night.

**Date: August 28, 2020**

**Time 7.30 AM**

Prisha’s vital signs are: HR 105, BP 130/80, Temp 37.3 C. Fetal HR is 180b/min with early decelerations.

Prisha has strong contractions, 5 in 10 and lasting 40 – 50 seconds each. She is fully dilated (10cm) and the fetus is cephalic in an Occiput-Anterior (OA) position. There is ++ caput and ++ moulding. Her membranes are intact. The doctor informs Prisha of the findings and asks to perform an artificial rupture of membranes (ARM) to assist with progress. Prisha provides her verbal consent to proceed with an ARM.

There is minimal amniotic fluid, and it has ++ mec.

**Time 7:31**

Prisha is moved onto her left-lateral side and she is given 500mL of IV fluids.

The fetal HR is now 155 b/min with early decelerations.

Prisha is requesting sips of water, which the midwife gives to her. Fetal monitoring is done every 5 minutes.

**Time 7:36**

Prisha continues to have strong, 5 in 10 contractions now lasting 60 seconds each.

Fetal HR is now 185 b/min with early decelerations. Prisha stands up and is moved to her right-hand side. Fetal HR lowers to 158 b/minute with positional change.

Prisha’s vital signs are: HR 110, BP 130/80, Temp 37.5C.

**Time 07:38**

Prisha now feels like she needs to push and is in the lithotomy position. The fetal head is on the perineum and appears to have significant caput +++ and molding ++ The Doctor protects Prisha’s perineum with a warm compress and pressure, and guide’s Prisha to push with contraction. The midwife communicates to the doctor that the fetal HR is now 90 with late decelerations.

**Time 07:39**

The fetal head is delivered, but shoulder dystocia is apparent.

The doctor tells Prisha that the baby’s body is difficult to deliver and that emergency procedures will need to be done, with the help of additional labour ward staff. The midwife calls the staff assist bell and then helps Prisha pull her knees towards her chest (McRobert’s) and tells Prisha that she is going to apply pressure to her abdomen to try and help the baby be delivered.

A live male infant is born at 07:40 vaginally. He is blue with poor tone. The umbilical cord is cut immediately to ensure neonatal resuscitation is performed. He cries with minimal ventilation. APGARS 4 and 8 at 1, and 5 mins. The infant is returned to the mother for skin-to-skin contact.

**LCG CLINCIAL CASE #4**

**Case 4.** Laura is 29-year-old G3P0 at 36+1 gestation. She reports vaginal loss for the last 5 hours. She has had an otherwise uncomplicated pregnancy with regular antenatal care. She has had 2 previous first trimester miscarriages.

**Date 31/08/2019**

**Time 23.20 pm**

The midwife makes an assessment in the admission room. She offers Laura a clinical evaluation. Laura accepts, and the midwife diagnosed that she is in active labour with regular, strong contractions (2 in 10 minutes), 5 cm cervical dilation, 3/5^th^ palpable cephalic, amniotic fluid with meconium +.

FHR is assessed and variable decelerations are observed, with return to baseline.

The midwife checks vital signs, and register that Laura has pulse 88, BP 120/85, Temperature 36.7C, P – A-.

**23:30** Laura is now in her room with her husband and is coping well with labour. She declines the offer of oral fluid, and she is lying in supine position in the bed.

**00:00** Laura is reassessed and continues with the same clinical parameters as at admission. The baseline FHR is 148, without decelerations. The frequency and duration of contractions are the same.

**00:30** She is accompanied by her husband; she is standing now and walking. She is tolerating contractions well and consumes water whenever she wishes. Continue with the same uterine dynamics. Baseline FHR 132, with variable dips. Amniotic fluid: meconium +. She is not receiving any medication.

**01.00** FHR 123, without decelerations. Movements felt. She was offered some pain relief and was given IV Tramadol.

**01.30** She is still accompanied by her husband, is in SP, tolerating contractions well and consumes water whenever she wants. DU 3/10/40 “, FHR 135, without decelerations.

**02:00** FHR 135 with no decelerations. She is sipping on water.

**02:30** She is very uncomfortable. Feels intense pain. Non-pharmacological analgesia is suggested for pain relief (relaxation technique). She accepts it. FHR130, no deceleration.

**03:00** She continues to have worsening abdominal and so the postgraduate doctor asks to perform a vaginal examination to assess progress. Laura provides consent. FHR 128, no deceleration. Contraction 3 in 10 minutes, duration: 40 second. Vaginal examination: 8cm dilation, 1/5^th^ palpable, cephalic, meconial amniotic fluid, caput +, moulding +

**03:30** FHR 130, variable decelerations. Nurse check vital signs. Laura continues with BP 130/85, pulse 92, Temperature 36.8.

**04:00** Laura advises the postgraduate doctor that she feels the urge to push. Her husband is next to her. FHR 126, with early decelerations. Laura consents to a vaginal examination which reveals she is fully dilated, station -2, LOT position, mec +, caput +, moulding +. Her contractions are 4 in 10, and lasting 50 seconds.

**04:30** Laura continues to push with the urge, and the FHR is checked every 5 minutes. There are no decelerations heard.

**05:05** Normal delivery, live fetus, birth weight 3730, Apgar 8/9,

**LCG Clinical Case #5**

**Case 5.** Maua Kasimu Rajabu, is a 38-year-old G4P2 who received limited antenatal care and has been induced yesterday (19^th^ July 2021) at 37 weeks due to Gestational Diabetes and high BMI. The midwives have called you for an assessment, as her Foley’s catheter has fallen out at 0500 this morning and she is asking for analgesia as she is in significant pain. Regular fetal and maternal assessments have been otherwise normal. Her sister, Maggie is accompanying her.

*Please complete your LCG using the clinical information provided.*

**20^th^ July 2021**

**11:27** The postgraduate doctor introduces herself to Maua and explains the need to perform a vaginal examination. Maua provides verbal consent. She is noticeably uncomfortable, contracting 4: 10, each lasting 40 seconds and so IV tramadol was administered prior to the examination. Maua is 8cm dilated, 1/5^th^ palpable, cephalic with membranes intact. FHR is 125 without any decelerations.

Maternal vital status: HR 97, BP 124/87, Temp 37.3 C, P-/A-

The postgraduate informs Maua of these findings and offers an artificial rupture of membrane (ARM).

**11:30:** ARM is performed, and amniotic fluid is noted to be pink (bloody). FHR remains 125 with no decelerations. Fetal movements are felt. Maua’s abdomen is soft in between contractions.

**11:32** Decision made for CTG due to blood-stained liqor and high-risk patient.

**11:45** CTG demonstrates a prolonged fetal bradycardia of 60 b/min. There is more blood on her pad. Patients reassess and is now full dilated, with fetal head on perineum. Maua is informed of the findings and told that a quick delivery is required.

Successful instrumental delivery of a liveborn male performed by the postgraduate doctor.

**11:46** Active management of third stage with delivery of placenta at 11:47. PPH of 800mL controlled by uteronics.

**LCG Clinical Case #6**

**Case 6.** Mrs Praatchi Patel is a 21-year-old G3P2 who presented to the hospital yesterday (20^th^ June 2021) with threatened preterm labour at 34+2 weeks gestation. She was given Nifedipine and morphine, and abdominal tightening stopped. This morning, her abdominal pain has started again and is now more intense. The nurse has paged you to review her.

- She has had 2 previous vaginal deliveries at term gestation
- This pregnancy, she had a small APH at 31+6 and was administered 2 x doses of Betamethasone (Celestone) at that time. No further bleeding and no cause of APH found.
- She is Rh Negative

*Please complete your LCG using the clinical information provided.*

**21^st^ June 2021 at 0550**

The postgraduate doctor introduces herself to Praachi who then takes a brief history. Praachi can feel the baby moving and has had no further PV loss. She is drinking small sips of water and has her sister present as her chosen birth companion. She explains her pain started around 0500 and has been increasing in frequency and intensity. She is worried the baby is going to come early.

The postgraduate doctor explains that she will need to perform maternal and fetal vital status assessment, as well as a vaginal examination to assess how dilated her cervix is and how far down the baby has come. Praatchi provides her verbal consent to proceed.

- FHR = 140, no decelerations
- Membranes intact
- Cervix = 5cm dilated
- Fetal position = cephalic
- Descent = 3/5^th^
- Caput/Moulding = N/A
- Contractions = 2:10 lasting 30 seconds
- Maternal vital signs = HR 110 b/min, BP 90/60, Temp 37.2 C
- Urine = P- / A-

**Q:** After your assessment of Praatchi at 0550, what would your next action and ongoing monitoring plan be? How do you discuss this with Praatchi?

Praachi is in labour and transferred to the labour ward for ongoing monitoring. A plan is made to re-examine her in 2 hours to determine labour progress, unless there are any abnormal maternal or fetal vital status signs.

**Supportive Care**

|  | **0600** | **0700** |
| --- | --- | --- |
| Companion | Yes | Yes |
| Pain Relief | Declined | Not Offered |
| Oral Fluid | Water | Decline |
| Posture | Supine | Supine |

**Baby**

|  | **05:30** | **06:00** | **06:30** | **07:00** | **07:30** |
| --- | --- | --- | --- | --- | --- |
| FHR | 157 | 138 | 143 | 153 | 154 |
| FHR Deceleration | N | N | N | N | N |

**Labour Progress**

The postgraduate doctor returns at 0800 as planned to reassess Praatchi and her labour progress. The postgraduate doctor asks for her consent, and Praachi agrees to be examined again.

*Please document the below findings onto your own LCG*

|  | **05:30** | **06:00** | **06:30** | **07:00** |
| --- | --- | --- | --- | --- |
| Contractions per 10 mins | 2 | 2 | 3 | 4 |
| Duration | 30 | 40 | 40 | 40 |

Maternal Vital Status: HR 125 b/min, BP 100/70, temp 37.6 C.

- FHR = 165 b/min with variable decelerations
- Cervix = 8cm dilated with membranes intact
- Cephalic
- 1/5^th^ palpable
- Caput + / Moulding –

**Q:** What would your plan to monitor her be? Do you have any concerns at this point?

*Please document the below findings onto your own LCG*

After discussion with Praatchi, a decision was made to perform an amniotomy at 0800. Amniotic was clear.

Her contractions were now strong, 4:10 lasting 40 seconds.

FHR after amniotomy was 167, and 15 minutes later at 0815 it was re-checked and was 170.

Praachi was placed on her left-lateral side and IV fluids were given to help with fetal tachycardia.

Praachi feels the urge to push. The fetal heart rate is measured every 5 minutes which continues to show a FHR of 164, 167 and 170 at 0820, 0825, 0830am respectively with variable decelerations. Maternal temperature was now 37.7 C. There are concerns of chorioamnionitis.

Praachi is re-examined at 0830 and the fetus is now at -2, LOA with caput ++ and moulding +

A liveborn female weighing 1850g was delivered at 0833 with Apgars of 6 and 8 at 1 and 5 minutes. She was transferred to the NICU for prematurity and treatment of presumed sepsis. Praachi was also given a course of antibiotics.

**LCG Clinical Case # 7**

**Case 7.** Bronte is a 28-year-old G1P0 at 35+2 presenting with premature rupture of membranes (13^th^ August 2021 at 0015). PV fluid was clear.

Vaginal examination was performed at 0300. Cervix was 2cm dilated and thick, 3/5^th^ palpable and cephalic. Amniotic fluid was clear. Maternal HR was 80 and temp 37.2 C. IV antibiotics commenced. Regular monitoring was performed and documented in the patient’s case file. You are called to see her at 0630 as she has started to have frequent abdominal pain and is requesting pain relief.

*Please complete your LCG using the clinical information provided.*

The postgraduate doctor introduced himself and discussed available pain relief options. Bronte has declined use of analgesia at this time. Repeat vaginal examination would be necessary, and Bronte gives consent for this to be performed.

**13^th^ August 2021 at 0630**

- Cervix = 5cm
- 3/5^th^ palpable
- Contracting 2: 10, lasting 30 seconds
- Amniotic fluid still leaking but remains clear.
- Maternal vital status = HR 88, BP 120/79, Temp 37.4 C. Urine A-/P-
- FHR = 135 with no decelerations.

**Q.** What is your clinical assessment and plan for continued monitoring of Bronte?

*Please discuss, and then continue to complete your LCG using the clinical information provided.*

Bronte is transferred to labour ward as she is now in the active first stage labour.

A plan is made to start an IV oxytocin infusion with reassessment in 4 hours (unless there are any abnormal maternal or infant vital signs, or concerns in clinical condition). The IV oxytocin infusion was started at 0700.

| **Supportive Care** | | | | | |
| --- | --- | --- | --- | --- | --- |
|  | **0630** | **0730** | **0830** | **0930** | **1030** |
| Companion | No | No | No | No | Declined |
| Pain Relief | Declined | Not Offered | Declined | Not offered | IV Tramadol |
| Oral Fluid | Water | Decline | Declined | Water | Water |
| Posture | Supine | Mobile | Supine | Mobile | Supine |

| **Baby** | | | | | |  |  |  |  |
| --- | --- | --- | --- | --- | --- | --- | --- | --- | --- |
|  | **06:30** | **07:00** | **07:30** | **08:00** | **08:30** | **09:00** | **09:30** | **10:00** | **10:30** |
| FHR | 145 | 133 | 147 | 151 | 160 | 157 | 158 | 163 | 165 |
| FHR Deceleration | N | V | N | N | N | N | N | N | V |

| **Labour Progress** | | | | | | | | | |
| --- | --- | --- | --- | --- | --- | --- | --- | --- | --- |
|  | **06:30** | **07:00** | **07:30** | **08:00** | **08:30** | **09:00** | **09:30** | **10:00** | **10:30** |
| Contractions per 10 mins | 2 | 2 | 3 | 3 | 3 | 3 | 3 | 3 | 4 |
| Duration | 30 | 30 | 30 | 40 | 40 | 40 | 50 | 40 | 40 |

**13^th^ August 2021 at 1030**

Bronte now asks for IV tramadol, which is administered. The IV oxytocin is now at 24 ml/hr.

Maternal vital signs: HR 90, BP 130/70, temp 37.5 C. Urine dipstick not performed.

- FHR 160 without decelerations
- Cervix = 7cm
- 2/5^th^ palpable
- OP

**Q.** What is your clinical assessment and plan for continued monitoring of Bronte?

*Please discuss, and then continue to complete your LCG using the clinical information provided.*

Findings were discussed with Bronte and a plan to continue IV syntocinon infusion and antibiotics, with review in 2 hours (unless otherwise indicated).

| **Supportive Care** | | |
| --- | --- | --- |
|  | **1130** | **1230** |
| Companion | Declined | No |
| Pain Relief | Declined | Not Offered |
| Oral Fluid | Water | Declined |
| Posture | Mobile | Supine |

| **Baby** | | | | | |
| --- | --- | --- | --- | --- | --- |
|  | **10:30** | **11:00** | **11:30** | **12:00** | **12:30** |
| FHR | 165 | 133 | 157 | 141 | 146 |
| FHR Deceleration | N | V | N | N | N |

| **Labour Progress** | | | | | |
| --- | --- | --- | --- | --- | --- |
|  | **10:30** | **11:00** | **11:30** | **12:00** | **12:30** |
| Contractions per 10 mins | 4 | 4 | 4 | 4 | 4 |
| Duration | 40 | 40 | 50 | 40 | 40 |

**13^th^ August 2021 at 12:30**

Maternal vital status = HR 110, BP 125/78, temp 37.4 C. Urine not collected.

- FHR 146 without decelerations
- OA with 1/5^th^ palpable
- Cervix = 10 cm
- Amniotic fluid remains clear.

Bronte starts actively pushing at 13:10, now 0/5^th^ palpable

|  | 1310 | 1315 | 1320 | 1325 | 1330 |
| --- | --- | --- | --- | --- | --- |
| FHR | 120 | 127 | 110 | 100 | 110 |
| FHR deceleration | N | N | V | V | V |
| Contractions per 10 mins | 4 | 4 | 5 | 5 | 5 |
| Duration of contractions | 50 | 50 | 50 | 50 | 50 |

Bronte is told that she will need a vacuum extraction and episiotomy, for which she agrees.

A live female infant is born, weighing 1.9kg and Apgars 6 and 9 at 1 and 5 minutes respectively. The baby is transferred to NICU due to prematurity and birthweight.

**LCG Case #8**

**Case 8.** Ms Catherine Basutu is a 30 year old G6P5 at 33+6 weeks gestation, who was referred to the high level obstetric hospital as her baby was diagnosed with a complex fetal cardiac anomaly at 20 week ultrasound. She had been booked for an induction of labour at 37 weeks but has ruptured her membranes at home this evening at 2250 and has come in with the husband. It is now 2315 on the 17^th^ September 2021. Fetal HR baseline is usually 90 -110.

She has had 5 previous vaginal deliveries at term, the last complicated by a PPH.

*Please complete your LCG using the clinical information provided.*

**17^th^ September 2021 at 2315**

The postgraduate doctor introduces herself to Catherine and explains that a vaginal examination would be required to assess progress. Catherine consents to this. She says that she was sleeping at home and woke up “wet”. She can feel the baby moving.

Maternal vital status signs = HR 100, BP 130/80, temp 37.2 C. Urine clear.

- FHR 100, no decelerations
- LOT
- Membranes ruptured, liqor clear.
- Cervix = 8cm dilated
- 1/5^th^ palpable

A CTG was placed to monitor baby and plan made to reassess Catherine in 2 hours unless there are any changes to her clinical condition.

| **Supportive Care** | | |
| --- | --- | --- |
|  | **2315** | **0015** |
| Companion | Yes | Yes |
| Pain Relief | Declined | Not Offered |
| Oral Fluid | Water | Declined |
| Posture | Mobile | Supine |

| **Baby** | | | | | |
| --- | --- | --- | --- | --- | --- |
|  | **2315** | **2345** | **0015** | **0045** | **0115** |
| FHR | 100 | 95 | 97 | 90 | 60 |
| FHR Deceleration | N | V | V | V | V |

| **Labour Progress** | | | | | |
| --- | --- | --- | --- | --- | --- |
|  | **2315** | **2345** | **0015** | **0045** | **0115** |
| Contractions per 10 mins | 4 | 4 | 4 | 4 | 4 |
| Duration | 40 | 40 | 50 | 40 | 40 |

You are called to urgently assess Catherine as the FHR is now 60 with variables. Her pain has increased, and she feels rectal pressure. The infant’s head is at the perineum when you assess her at 0117. The baby is delivered at 0120 and makes no respiratory effort. Pediatric resuscitation is performed and continued for 30 mins. Resuscitation is discontinued after discussion with the family.

*Please discuss, and then continue to complete your LCG using the clinical information provided.*
